# A decade of progress and challenges in Water, Sanitation and Hygiene (WASH) coverage in Bangladesh: Insights from Bangladesh demographic and health survey 2011–2022

**DOI:** 10.1371/journal.pone.0354480

**Published:** 2026-07-23

**Authors:** Md. Hasibul Islam Jitu, Marufa Jahan, Syed Shahadat Hossain

**Affiliations:** 1 Institute of Statistical Research and Training, University of Dhaka, Dhaka, Bangladesh; 2 IHE Delft Institute for Water Education, Delft, Netherlands; World Health Organization, Regional Office for South-East Asia, INDIA

## Abstract

**Background:**

Access to safe water, sanitation, and hygiene (WASH) remains a critical global public health challenge, with an estimated 2 billion people lacking safe drinking water and basic hygiene services. In Bangladesh, disparities in WASH access persist across regions and socioeconomic groups. This study assesses decade-long changes in WASH coverage, examines its distribution across socio-demographic groups, identifies key associated factors, and highlights underserved regions and population subgroups.

**Methods:**

Data from four rounds of the Bangladesh Demographic and Health Survey (BDHS) (2011–2022) were analyzed. WASH indicators were classified as improved or unimproved based on WHO–UNICEF Joint Monitoring Programme (JMP) 2017 guidelines and applied consistently across all survey rounds. Spatial mapping was used to assess regional disparities, while regression models identified factors associated with WASH facilities. A classification tree approach was used to explore interactions among predictors.

**Results:**

Over the decade (2011–2022), access to improved drinking water remained high (above 98%), with improvements in sanitation (22 percentage points), hygiene (30.5 percentage points), and overall WASH facilities (28 percentage points). Classification tree analysis identified wealth index as the most important determinant. Regional disparities were evident, with higher coverage in Dhaka, Rajshahi, and Chattogram, and lower coverage in Barishal and Mymensingh. Wealthier households had higher odds of improved WASH facilities (AOR: 8.72 [6.74, 11.3] in 2011; 9.35 [8.31, 10.5] in 2022) compared to poorer households. Households with more higher educated heads also had higher odds (AOR: 7.10 [5.80, 8.69] in 2011; 3.44 [2.99, 3.96] in 2022) compared to those with no formal education. Other associated factors included family size, region, media access, and mobile ownership.

**Conclusion:**

Key determinants of WASH access, including wealth, education, and regional disparities, remained largely unchanged over time. Achieving SDG 6 requires targeted subsidies for socioeconomically disadvantaged households, region-specific interventions in underserved areas such as Barishal and Mymensingh, and strengthened awareness programs.

## Introduction

Access to safe and adequate water, sanitation, and hygiene (WASH) is essential for improving public health, advancing economic growth, and enhancing overall well-being [1,2]. Recognizing its importance, the United Nations (UN) declared access to water and sanitation a human right in 2010 [3]. In 2015, the UN included WASH into Sustainable Development Goal (SDG) 6, aiming for universal access to safe and reliable water and sanitation services by 2030 [4]. Before being incorporated into the SDGs, WASH was part of the Millennium Development Goals (MDGs), specifically target 7c, which aimed to ensure sustainable access to safe drinking water and basic sanitation by 2015 [5]. While the MDG target for drinking water was met, sanitation target lagged behind [6].

Despite global commitments, SDG 6 also remains off track, with the COVID-19 pandemic further highlighted how inadequate water, sanitation, and hygiene (WASH) services deepen health and social inequalities [7–9]. Additional challenges such as climate change, rapid urbanization, humanitarian crises, and persistent socioeconomic disparities further worsen the situation, leaving nearly half the world without access to safe sanitation in 2020 [3]. According to the Joint Monitoring Programme (JMP), over 2 billion people lack access to safe drinking water, 2 billion lack handwashing facilities, and 419 million still practice open defecation [10]. In South Asia, over 134 million people still lack safe drinking water, with contamination levels in water sources ranging from 68% to 84% [11].

In response to these ongoing challenges, the UN-Water Global Analysis and Assessment of Sanitation and Drinking-Water (GLAAS) Strategy (2023–2030) prioritizes strengthening WASH systems through improved monitoring, data accessibility, and policy reforms. Key focus areas include climate resilience, gender inclusion, digital tools, and global collaboration to ensure sustainable water and sanitation access [12]. However, low- and middle-income countries (LMICs) like Bangladesh continue to struggle with WASH challenges. While Bangladesh has nearly eradicated open defecation (98.5%), only 31% of rural areas have access to safely managed sanitation, according to the JMP 2017 report [13]. Further studies indicate that 15.4% of households lack improved sanitation, 43.72% lack improved hygiene facilities, and 48.72% lack access to improved WASH services [14].

In the context of SDG 6, Bangladesh shows differing levels of progress across WASH components compared with other LMICs, particularly in South Asia. A recent comparative analysis across Afghanistan, Bangladesh, Nepal, and Pakistan reported that overall WASH coverage in Bangladesh was 50.3%, which is lower than Nepal (75.3%) and Pakistan (59.5%), but higher than Afghanistan (33.5%) [14]. Bangladesh has achieved very high access to improved drinking water, exceeding 98% coverage; however, this progress is not consistently reflected in sanitation and hygiene services, where coverage remains comparatively lower [14]. Similar patterns have been reported in other LMICs, where progress in drinking water access has outpaced improvements in sanitation and hygiene services [8]. According to the WHO/UNICEF Joint Monitoring Programme (JMP), access to safely managed sanitation services in Bangladesh remains below 40% and lower than global averages [8].

The consequences of inadequate WASH are severe. Poor conditions contribute to communicable diseases such as diarrhea, with 88% of cases linked to contaminated water, inadequate sanitation, and poor hygiene [14–16]. Beyond immediate health risks, inadequate WASH leads to poor educational outcomes, child malnutrition, vulnerability to disasters, increased child mortality, and rising healthcare costs [1,4,14,17]. Notably, improving WASH practices could prevent 2.4 million deaths annually, underscoring the urgent need for global efforts to address this challenge [15,16].

To better understand WASH progress and investment trends, the Bangladesh Bureau of Statistics (BBS), with support from WaterAid Bangladesh, launched the first-ever ‘National WASH Accounts 2020’ report on 2 November 2023 [18]. This report provides insights into national WASH expenditure, revealing that in 2020, total WASH expenditure amounted to BDT 598 billion (2.18% of GDP), with BDT 172 billion (28%) allocated for capital investment in infrastructure and services and over 60% spending on ensuring adequate hygiene activities [19]. Households spent an average of BDT 11,574 annually on WASH, which accounted for 4.3% of their annual income. Notably, low-income households dedicated a substantial portion of their earnings to these essential services [18,19].

Research has identified significant demographic, socioeconomic, and regional disparities in WASH coverage across Bangladesh and other LMICs in South Asia [14,15,20–22]. Many studies have examined factors associated with WASH and its association with various health outcomes at local and national levels [14,15,20,23–25]. Key influencing factors include geographic location, economic constraints, household environment, income, ethnicity, education, infrastructure availability, and cultural beliefs [22,26–28]. In addition, broader national assessments have attempted to document long-term progress and challenges in Bangladesh [29]. However, there remains a lack of integrated analyses that simultaneously examine temporal changes, socio-demographic distribution, associated factors, and high-risk population subgroups within a unified framework using nationally representative data in Bangladesh.

To address this gap, this study aims to assess decade-long changes in WASH coverage, examine its distribution across socio-demographic groups, identify key factors associated with WASH access, and highlight underserved regions and population subgroups using four rounds of nationally representative Bangladesh Demographic and Health Surveys (BDHS) data from 2011 to 2022.

## Methods

### Data sources

**Fig 1:**
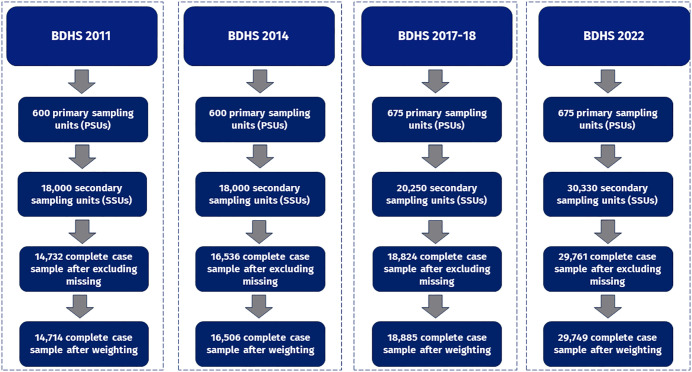
Datasets used in this study.

The Bangaldesh Demographic and Health Survey (BDHS) data from four survey rounds: 2011 (n = 14,714), 2014 (n = 16,506), 2017−18 (n = 18,885), and 2022 (n = 29,749) serves as the data source for this study [30]. These four survey periods were selected to compare WASH facilities progress and explore changes in associated sociodemographic factors over past decade. The BDHS surveys employed a two-stage stratified sampling strategy to ensure a nationally representative sample. In the first stage, Primary Sampling Units (PSUs) or enumeration areas (EA) were selected with probability proportional to EA size. In the second stage, a specific number of Secondary Sampling Units (SSUs) or households were systematic sampled within each PSU. This approach ensured the representation of both urban and rural areas. Each survey followed standardized protocols and received approval from the respective authorities. **Fig 1** illustrated the process of deriving the final sample used in this study.

### Outcome variables and covariates

The study considered WASH as the primary outcome variable, evaluating the overall status of three domains: household drinking water, sanitation, and hygiene facilities. According to the WHO-UNICEF JMP 2017 guidelines, these domains were classified as improved or unimproved [13]. Drinking water facilities were categorized into improved (e.g., piped water into dwelling/yards/plot/neighbor; water from public tap/standpipe; water from tube well or borehole; water from protected dug well, protected springs; rainwater; tanker truck/cart with small tank; bottled water) and unimproved (e.g., surface water; unprotected dug well; unprotected spring and other unreliable sources). Similarly, sanitation facilities were classified as improved (e.g., flush or pour-flush toilets connected to piped sewer system/septic tanks/pit latrines/don’t know where; pit latrines with slab; composting toilet) and unimproved (e.g., flush or pour-flush toilets not connected to piped sewer system/septic tanks/pit latrines; pit latrine without slab/open pit bucket; hanging toilet/hanging latrine and other inadequate sanitation facilities). Hygiene facilities was categorized as improved based on the availability of observed handwashing place equipped with water and soap/detergent otherwise categorized as unimproved. Then WASH facilities variable was considered as improved when all of these three domains (drinking water, sanitation, and hygiene facilities) were improved otherwise considered as unimproved [14,15].

The study considers several sociodemographic variables as covaraies, including area of residence (urban, rural), region (Dhaka, Barishal, Mymensingh, Chattogram, Sylhet, Khulna, Dhaka, Rajshahi, and Rangpur), sex of the household head (male, female), age of the household head (<35, 35–54, and 54+) [31], education of the household head (no education or pre-school, primary, secondary, and higher), family size (1–4, 5 + members) [15], wealth index (poor, middle, and rich), mass media accessibility (with access, without access) and owning mobile phone (yes, no). Access to mass media was determined by the availability of radio or television [14], The five wealth index quintiles were reclassified into three categories: ‘Poor’ (combining ‘Poorest’ and ‘Poorer’), ‘Middle,’ and ‘Rich’ (combining ‘Richer’ and ‘Richest’), as adopted from Dhital et al. (2022) [32]. In 2015, the Dhaka division was divided into Dhaka and Mymensingh. Consequently, surveys conducted before that, such as BDHS 2011 and 2014, considered seven regions, whereas later surveys, including BDHS 2017–18 and 2022, considered eight regions.

**Fig 2:**
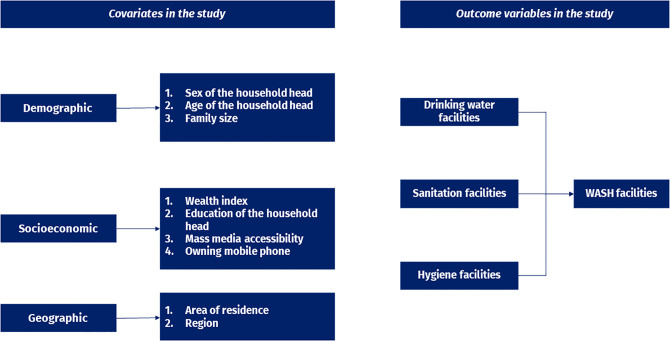
Outcome variables and covariates of this study.

**Fig 2** illustrated the outcome variables and covariates of this study.

### Statistical analyses

Since this study utilized large-scale survey data, missing cases were handled through listwise deletion under the assumption that the data were missing at random [33,34]. All analyses were conducted separately for each survey round, while accounting for the complex survey design through the incorporation of sampling weights, clustering at the primary sampling unit (PSU) level, and stratification using the survey package in R. Survey-weighted descriptive analyses were conducted to provide an overview of the data, with weighted frequencies and percentages used to describe the distribution of WASH facilities across demographic, socio-economic, and geographic factors. The Average Annual Rate of Increase (AARI) was calculated to quantify changes in WASH coverage over time using survey-weighted proportions. AARI was defined as the geometric annual rate of change between survey periods, following established approaches in demographic and public health research [35]. Both interval-specific (2011–2014, 2014–2017/18, and 2017/18–2022) and overall (2011–2022) estimates were computed to assess temporal trends in WASH coverage across demographic, socio-economic, and geographic factors.

Geographical mapping was based on raw survey-weighted proportions of WASH coverage at the regional level to illustrate spatial variation and changes over time. No spatial smoothing techniques or formal statistical tests for spatial clustering (e.g., Moran’s I) were applied, and the maps are presented for descriptive purposes only. Additionally, survey-weighted logistic regression models with a quasi-binomial distribution were applied to identify key factors associated with WASH while accounting for overdispersion. Multicollinearity among predictors in the fitted logistic regression models was assessed using Generalized Variance Inflation Factor (GVIF) scores. Because several predictors were categorical with multiple levels, squared adjusted GVIF values were used to allow comparison across variables with different degrees of freedom. Squared adjusted GVIF values below 5 were considered indicative of no serious multicollinearity [36].

To capture complex predictor–outcome interactions, classification tree analysis was employed using the overall WASH facilities variable as the outcome. This method partitions the data through hypothesis testing at each node, selecting the predictor with the strongest association based on p-values. Survey weights were incorporated as case weights during tree construction to account for unequal probability of selection. The splitting process continued only when statistically significant relationships were identified, based on a predefined significance threshold (*mincriterion*), which helped control tree growth and reduce the risk of overfitting. Terminal nodes summarize the distribution of the outcome variable within each segment [37].

The study utilized statistical software R (version 4.4.1) for all statistical analyses and geographic mapping [38]. Key R packages used included ggplot2 [39], gridExtra [40], car [41], partykit [37], survey [42], tidyverse [43]. From an openly licensed source ‘The geoBoundaries Global Administrative Database’, the administrative boundary shapefiles for Bangladesh were obtained [44].

### Ethical considerations

No ethical approval was required for this study as we utilized publicly available secondary data from BDHS, which had been already approved by institutional review boards (IRBs) at ICF and the Bangladesh Medical Research Council (BMRC). Additionally, written informed consent was obtained from participants before data collection, ensuring voluntary participation, confidentiality, and the right to withdraw at any time. The datasets were fully anonymized before access and are available upon request for research purposes through the DHS website (https://dhsprogram.com/).

## Results

### Coverage of WASH facilities

**Fig 3:**
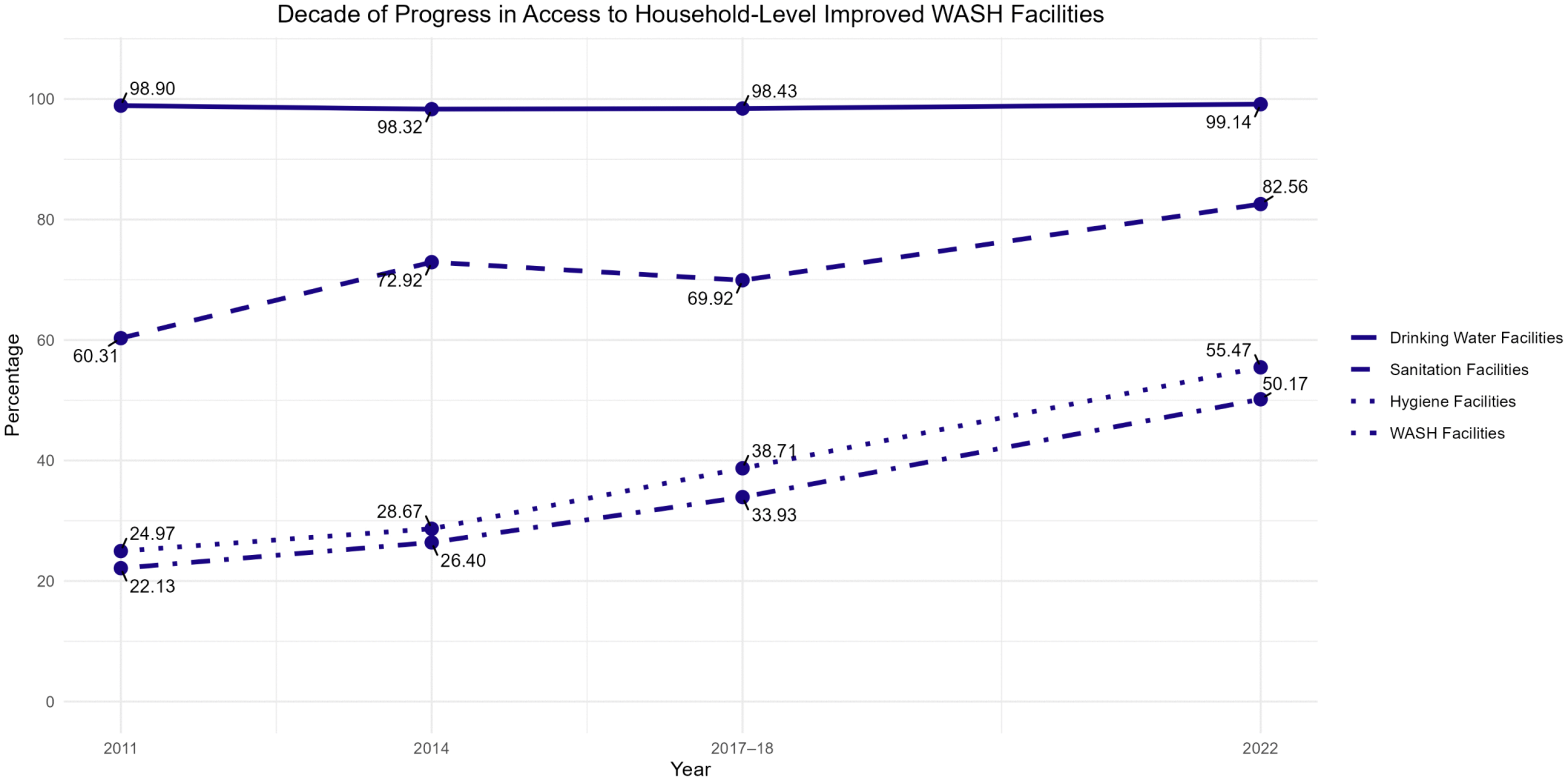
Decade of progress in access to household-level improved WASH facilities in Bangladesh.

**Fig 3** illustrated the decade-long progress in improved drinking water, sanitation, hygiene, and overall WASH facilities in Bangladesh While access to drinking water remained consistently high at over 98%, sanitation facilities increased by 22%, from 60.31% in 2011 to 82.56% in 2022. However, improved hygiene facilities were significantly lower in 2011, with only 24.97% coverage. While this improved over the decade, 44.53% of households still lacked access to adequate hygiene facilities. Similarly, improved WASH facilities were limited in 2011 (22.13%) and showed progress over time; however, 49.83% of households still lacked access to improved WASH facilities in 2022.

### Region-wise household level coverage of WASH facilities

**Fig 4:**
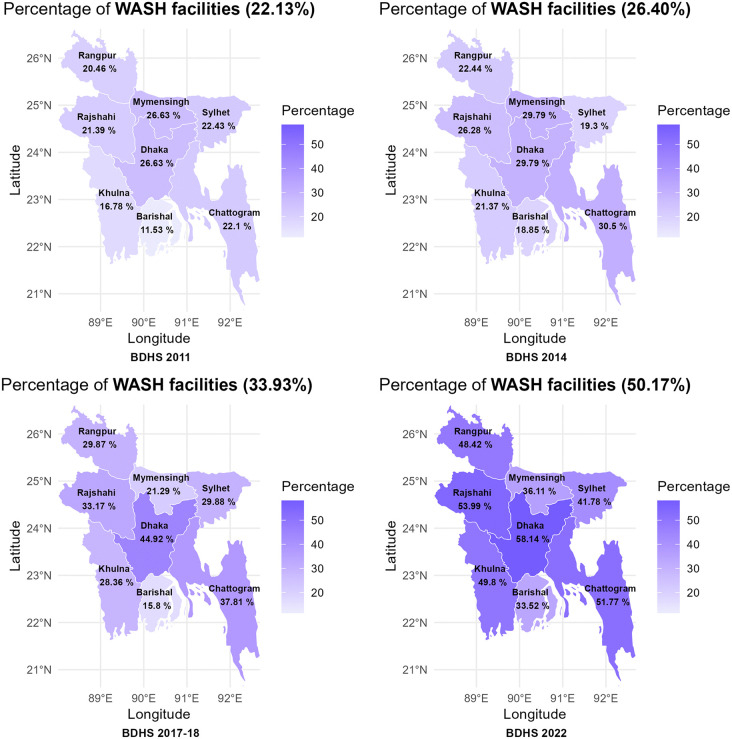
Region-wise household level coverage of WASH facilities in Bangladesh across four survey periods.

**Fig 4** showed the percentage of households with access to improved WASH facilities across different regions of Bangladesh over four survey periods (2011, 2014, 2017−18, and 2022). Over the decade, there has been a clear trend of increasing WASH coverage across almost all regions, with the national average rising from 22.13% in 2011 to 50.17% in 2022.

However, regional disparities persist. Some regions, such as Dhaka, Rajshahi, and Chattogram, consistently show higher WASH coverage, while Barishal and Mymensingh lag behind in most survey years. Progress is particularly notable in Rajshahi, where coverage increased from 21.39% in 2011 to 53.99% in 2022, and in Dhaka, which reached 58.14% in 2022 from 26.63% in 2011. Khulna also showed significant improvement, rising from 16.78% in 2011 to 49.8% in 2022.

In contrast, Barishal had the lowest coverage in 2011 (11.53%) and, despite improvement, remained as the least covered regions in 2022 (33.52%). Mymensingh also performed poorly in the most two surveys, with only 15.8% and 36.11% improved WASH coverage respectively in 2017−18 and 2022.

### Distribution of WASH facilities by demographic, socio-economic, and geographic factors

The percentage distribution of households with WASH facilities in Bangladesh across four survey periods is illustrated in Table 1. Urban households had the highest WASH coverage in all survey years, while rural households consistently had the lowest. Urban households had a notable increase in access to improved WASH facilities, rising from 44.74% in 2011 to 65.56% in 2022. Rural households also improved, though at a higher rate, from 13.71% in 2011 to 44.09% in 2022. Rich household consistently having the highest WASH coverage across all periods, while the poor households had the lowest coverage. In 2011, only 3.12% of the poor households had access to improved WASH facilities, which increased to 23.04% in 2022. Meanwhile, the richest households observed an increase from 45.51% in 2011 to 79.12% in 2022. There are no notable variations in WASH coverage among household headed by male and female. However, both sexes observed similar improvements over the decade. Across all survey years, households with higher educated household heads had the highest WASH coverage, while those with no education had the lowest. Households led by individuals with no education improving from 7.59% in 2011 to 34.85% in 2022, whereas those with higher education saw an increase from 61.60% to 82.68%. Further, households headed by middle and older individuals (35–54 and 54+) had higher coverage across all survey years, while younger-headed (<35) households had lower coverage. Households headed by individuals aged 54 + increased WASH coverage from 23.84% in 2011 to 49.93% in 2022, whereas those headed by younger individuals observed an increase from 17.69% to 46.05%. Similarly, smaller family size (1–4 members) improving from 20.84% to 48.81%, while larger families (4 + members) increasing only from 23.41% to 52.17% over the decade. Despite improvements, smaller households consistently had lower WASH coverage, whereas larger families had higher coverage. Households with mass media access had higher WASH coverage, while those without media access had the lowest coverage across all survey years. Besides, households accessed to mass-media improving WASH coverage from 38.44% in 2011 to 63.93% in 2022, compared to those without access, which improved only from 7.92% to 36.08%. Mobile phone-owning households had consistently higher WASH coverage across the years, while those without mobile phones had the lowest. Households owning mobile phones saw an increase from 26.41% in 2011 to 50.92% in 2022, while those without mobile phones remained significantly lower, increasing only from 4.65% to 15.34%.

**Table 1:** The frequency distribution of WASH facilities by demographic, socio-economic, and geographic factors.

Variables	BDHS 2011		BDHS 2014		BDHS 2017−18		BDHS 2022	
	Total sample, N = 14,714 (%)	N (%) of household with improved WASH facilities	Total sample, N = 16,506 (%)	N (%) of household with improved WASH facilities	Total sample, N = 18,885 (%)	N (%) of household with improved WASH facilities	Total sample, N = 29,749 (%)	N (%) of household with improved WASH facilities
**Area of residence**								
Urban	3,994 (27.14)	1,787 (44.74)	4,751 (28.79)	2,217 (46.66)	5,431 (28.76)	2,921 (53.79)	8,437 (28.36)	5,531 (65.56)
Rural	10,721 (72.86)	1,470 (13.71)	11,755 (71.21)	2,141 (18.21)	13,454 (71.24)	3,486 (25.91)	21,312 (71.64)	9,396 (44.09)
**Region**								
Dhaka	4,898 (33.29)	1,304 (26.63)	5,838 (35.37)	1,739 (29.79)	4,874 (25.81)	2,189 (44.92)	7,618 (25.61)	4,429 (58.14)
Barishal	759 (5.16)	87 (11.53)	971 (5.88)	183 (18.85)	1,009 (5.34)	159 (15.80)	1,768 (5.94)	593 (33.52)
Chattogram	2,427 (16.50)	536 (22.10)	2,877 (17.43)	877 (30.50)	3,182 (16.85)	1,203 (37.81)	5,124 (17.23)	2,653 (51.77)
Khulna	1,711 (11.63)	287 (16.78)	1,748 (10.59)	374 (21.37)	2,217 (11.74)	629 (28.36)	3,501 (11.77)	1,744 (49.80)
Rajshahi	2,197 (14.93)	470 (21.39)	2,057 (12.46)	541 (26.28)	2,704 (14.32)	897 (33.17)	4,121 (13.85)	2,225 (53.99)
Rangpur	1,988 (13.51)	407 (20.46)	1,976 (11.97)	443 (22.44)	2,359 (12.49)	705 (29.87)	3,560 (11.97)	1,724 (48.42)
Sylhet	734 (4.99)	165 (22.43)	1,040 (6.30)	201 (19.30)	974 (5.16)	291 (29.88)	1,677 (5.64)	701 (41.78)
Mymensingh					1,566 (8.29)	334 (21.29)	2,379 (8.00)	859 (36.11)
**Wealth index**								
Poor	5,688 (38.66)	177 (3.12)	6,447 (39.06)	280 (4.34)	7,576 (40.12)	592 (7.81)	12,165 (40.89)	2,802 (23.04)
Middle	2,830 (19.23)	260 (9.18)	3,273 (19.83)	465 (14.20)	3,720 (19.70)	1,006 (27.03)	5,968 (20.06)	2,933 (49.14)
Rich	6,196 (42.11)	2,820 (45.51)	6,785 (41.11)	3,613 (53.25)	7,588 (40.18)	4,809 (63.38)	11,616 (39.05)	9,191 (79.12)
**Sex of the household head**								
Male	13,101 (89.04)	2,890 (22.06)	14,450 (87.54)	3,817 (26.41)	15,904 (84.22)	5,422 (34.09)	25,038 (84.17)	12,662 (50.57)
Female	1,613 (10.96)	367 (22.72)	2,056 (12.46)	541 (26.32)	2,980 (15.78)	984 (33.02)	4,711 (15.83)	2,264 (48.07)
**Age of the household head**								
<35	3,702 (25.16)	655 (17.69)	4,186 (25.36)	855 (20.42)	4,375 (23.17)	1,334 (30.49)	5,928 (19.93)	2,730 (46.05)
35-54	7,173 (48.75)	1,686 (23.51)	7,832 (47.45)	2,235 (28.54)	9,013 (47.73)	3,192 (35.41)	14,642 (49.22)	7,613 (51.99)
54+	3,839 (26.09)	915 (23.84)	4,489 (27.19)	1,268 (28.25)	5,496 (29.11)	1,881 (34.22)	9,179 (30.86)	4,583 (49.93)
**Education of the household head**								
No education	5,252 (35.69)	399 (7.59)	5,618 (34.03)	570 (10.15)	5,364 (28.41)	1,000 (18.64)	9,152 (30.76)	3,190 (34.85)
Primary	4,008 (27.24)	596 (14.87)	4,650 (28.17)	866 (18.62)	6,233 (33.00)	1,631 (26.17)	8,488 (28.53)	3,688 (43.44)
Secondary	3,661 (24.88)	1,157 (31.61)	4,309 (26.10)	1,583 (36.73)	4,920 (26.05)	2,096 (42.60)	8,420 (28.30)	5,000 (59.38)
Higher	1,793 (12.19)	1,105 (61.60)	1,929 (11.69)	1,339 (69.40)	2,367 (12.54)	1,679 (70.93)	3,689 (12.40)	3,050 (82.68)
**Family size**								
1-4	7,323 (49.77)	1,526 (20.84)	8,770 (53.13)	2,206 (25.16)	10,632 (56.30)	3,456 (32.50)	17,675 (59.41)	8,628 (48.81)
4+	7,392 (50.23)	1,731 (23.41)	7,737 (46.87)	2,152 (27.81)	8,252 (43.70)	2,951 (35.76)	12,074 (40.59)	6,299 (52.17)
**Mass media accessibility**								
With access	6,851 (46.56)	2,634 (38.44)	7,606 (46.08)	3,476 (45.70)	9,176 (48.59)	4,731 (51.56)	15,054 (50.60)	9,625 (63.93)
Without access	7,863 (53.44)	623 (7.92)	8,901 (53.92)	882 (9.91)	9,709 (51.41)	1,676 (17.26)	14,695 (49.40)	5,302 (36.08)
**Owning mobile phone**								
Yes	11,822 (80.34)	3,122 (26.41)	14,685 (88.97)	4,269 (29.07)	17,859 (94.57)	6,303 (35.29)	29,127 (97.91)	14,831 (50.92)
No	2,893 (19.66)	134 (4.65)	1,821 (11.03)	89 (4.89)	1,025 (5.43)	104 (10.12)	622 (2.09)	95 (15.34)

### Average Annual Rate of Increase (AARI)

Table 2 presents the Average Annual Rate of Increase (AARI) in improved WASH coverage across demographic, socio-economic, and geographic characteristics in Bangladesh between 2011 and 2022. Overall, WASH coverage increased across all population subgroups, although the rate of improvement varied considerably.

**Table 2:** Average Annual Rate of Increase (AARI) of improved WASH facilities by demographic, socio-economic, and geographic factors in Bangladesh, 2011–2022.

Variables	BDHS 2011: Improved WASH (%)	AARI 2011–2014 (%)	BDHS 2014: Improved WASH (%)	AARI 2014–2017/18 (%)	BDHS 2017−18: Improved WASH (%)	AARI 2017/18–2022 (%)	BDHS 2022: Improved WASH (%)	AARI 2011–2022 (%)
**Area of residence**								
Urban	44.74	1.41	46.66	3.62	53.79	5.07	65.56	3.53
Rural	13.71	9.92	18.21	9.22	25.91	14.21	44.09	11.20
**Region**								
Dhaka	26.63	3.81	29.79	10.81	44.92	6.66	58.14	7.36
Barishal	11.53	17.80	18.85	−4.32	15.80	20.69	33.52	10.19
Chattogram	22.10	11.34	30.50	5.52	37.81	8.17	51.77	8.05
Khulna	16.78	8.39	21.37	7.33	28.36	15.11	49.80	10.39
Rajshahi	21.39	7.10	26.28	5.99	33.17	12.95	53.99	8.78
Rangpur	20.46	3.13	22.44	7.41	29.87	12.84	48.42	8.15
Sylhet	22.43	−4.89	19.30	11.55	29.88	8.74	41.78	5.82
Mymensingh					21.29	14.12	36.11	
**Wealth index**								
Poor	3.12	11.63	4.34	15.82	7.81	31.06	23.04	19.93
Middle	9.18	15.65	14.20	17.46	27.03	16.12	49.14	16.48
Rich	45.51	5.38	53.25	4.45	63.38	5.70	79.12	5.16
**Sex of the household head**								
Male	22.06	6.18	26.41	6.59	34.09	10.36	50.57	7.83
Female	22.72	5.02	26.32	5.83	33.02	9.84	48.07	7.05
**Age of the household head**								
<35	17.69	4.90	20.42	10.54	30.49	10.86	46.05	9.09
35-54	23.51	6.68	28.54	5.54	35.41	10.08	51.99	7.48
54+	23.84	5.82	28.25	4.91	34.22	9.91	49.93	6.95
**Education of the household head**								
No education	7.59	10.17	10.15	16.41	18.64	16.93	34.85	14.86
Primary	14.87	7.78	18.62	8.88	26.17	13.51	43.44	10.24
Secondary	31.61	5.13	36.73	3.78	42.60	8.66	59.38	5.90
Higher	61.60	4.05	69.40	0.55	70.93	3.91	82.68	2.71
**Family size**								
1-4	20.84	6.48	25.16	6.61	32.50	10.70	48.81	8.04
4+	23.41	5.91	27.81	6.49	35.76	9.90	52.17	7.56
**Mass media accessibility**								
With access	38.44	5.94	45.70	3.06	51.56	5.52	63.93	4.73
Without access	7.92	7.76	9.91	14.88	17.26	20.24	36.08	14.78
**Owning mobile phone**								
Yes	26.41	3.25	29.07	4.97	35.29	9.60	50.92	6.15
No	4.65	1.69	4.89	19.94	10.12	10.96	15.34	11.46

Substantial gains were observed among disadvantaged groups. Rural households experienced a higher annual increase (AARI = 11.20%) compared to urban households (3.53%). Similarly, the poorest households showed the fastest improvement (AARI = 19.93%), followed by middle-income households (16.48%), while the richest group exhibited comparatively slower growth (5.16%). Higher rates of increase were also observed among households without access to mass media (14.78%) and those without mobile phones (11.46%).

Regional variation in AARI was evident. Khulna (10.39%) and Barishal (10.19%) demonstrated relatively higher long-term growth, whereas Sylhet showed comparatively slower progress (5.82%). Temporal patterns further indicate fluctuations in growth across survey intervals, with some regions experiencing short-term declines or slower growth during specific periods.

Across education levels, households with no formal education exhibited the highest rate of improvement (14.86%), while those with higher education showed more modest increases (2.71%). Similar patterns were observed across age groups and sex of household head, where improvements were relatively consistent, with slightly higher rates among younger household heads.

Supplementary S1 Table presents the AARI for individual WASH components. The overall AARI for improved WASH coverage was 7.72% over the study period. The improvements were most pronounced for hygiene (7.53%) and overall WASH (7.72%), followed by sanitation (2.90%), while access to improved drinking water remained nearly constant (0.02%) due to already high baseline coverage.

### Binary logistic regression model

The findings of a binary logistic regression model using WASH facilities as outcome for each of the four survey is displayed in **Table 3**. Compared to urban households, rural households were associated with significantly lower odds of accessing improved WASH facilities in earlier survey years, with 52% lower odds in 2011 (AOR: 0.48 [95% CI: 0.40, 0.57]; p < 0.001), 36% lower odds in 2014 (AOR: 0.64 [95% CI: 0.54, 0.77]; p < 0.001), and 22% lower odds in 2017–18 (AOR: 0.78 [95% CI: 0.67, 0.90]; p = 0.001). However, this association was no longer significant in 2022. Compared to the Dhaka region, the odds of improved WASH access were lower in Barishal across all years, with AOR: 0.56 (95% CI: 0.41, 0.75; p < 0.001) in 2011, AOR: 0.66 (95% CI: 0.43, 1.00; p = 0.050) in 2014, AOR: 0.42 (95% CI: 0.32, 0.54; p < 0.001) in 2017–18, and AOR: 0.72 (95% CI: 0.57, 0.90; p = 0.005) in 2022. In contrast, households form Rajshahi were associated with higher odds of accessing improved WASH facilities compared to the Dhaka across all survey years, with AOR: 1.54 (95% CI: 1.18, 2.01; p = 0.002) in 2011, AOR: 1.68 (95% CI: 1.26, 2.25; p < 0.001) in 2014, AOR: 1.32 (95% CI: 1.04, 1.69; p = 0.025) in 2017–18, and AOR: 1.58 (95% CI: 1.28, 1.95; p < 0.001) in 2022. Similarly, households form Rangpur were also associated with higher odds of accessing improved WASH facilities compared to the Dhaka across all years, with AOR: 2.58 (95% CI: 1.94, 3.43; p < 0.001) in 2011, AOR: 1.89 (95% CI: 1.24, 2.88; p = 0.003) in 2014, AOR: 1.91 (95% CI: 1.49, 2.45; p < 0.001) in 2017–18, and AOR: 1.85 (95% CI: 1.49, 2.29; p < 0.001) in 2022.

**Table 3:** Binary logistic regression model adjusted for demographic, socio-economic, and geographic factors with WASH facilities as outcome.

Variables	BDHS 2011			BDHS 2014			BDHS 2017−18			BDHS 2022		
	AOR	95% CI	p-value	AOR	95% CI	p-value	AOR	95% CI	p-value	AOR	95% CI	p-value
**Area of residence**												
Urban (ref.)	—	—		—	—		—	—		—	—	
Rural	0.48	0.40, 0.57	**<0.001**	0.64	0.54, 0.77	**<0.001**	0.78	0.67, 0.90	**0.001**	1.02	0.88, 1.19	0.75
**Region**												
Dhaka (ref.)	—	—		—	—		—	—		—	—	
Barishal	0.56	0.41, 0.75	**<0.001**	0.66	0.43, 1.00	0.050	0.42	0.32, 0.54	**<0.001**	0.72	0.57, 0.90	**0.005**
Chattogram	0.97	0.75, 1.24	0.78	1.11	0.83, 1.47	0.49	1.00	0.79, 1.27	0.97	1.43	1.14, 1.80	**0.002**
Khulna	0.72	0.55, 0.95	**0.020**	0.81	0.61, 1.08	0.16	0.70	0.55, 0.90	**0.006**	0.94	0.74, 1.19	0.59
Rajshahi	1.54	1.18, 2.01	**0.002**	1.68	1.26, 2.25	**<0.001**	1.32	1.04, 1.69	**0.025**	1.58	1.28, 1.95	**<0.001**
Rangpur	2.58	1.94, 3.43	**<0.001**	1.89	1.24, 2.88	**0.003**	1.91	1.49, 2.45	**<0.001**	1.85	1.49, 2.29	**<0.001**
Sylhet	1.29	0.99, 1.68	0.062	1.03	0.76, 1.41	0.83	0.89	0.68, 1.18	0.43	0.88	0.69, 1.13	0.32
Mymensingh							0.77	0.58, 1.01	0.056	1.00	0.81, 1.23	0.96
**Wealth index**												
Poor (ref.)	—	—		—	—		—	—		—	—	
Middle	1.91	1.47, 2.48	**<0.001**	1.96	1.58, 2.43	**<0.001**	3.34	2.86, 3.89	**<0.001**	2.81	2.56, 3.09	**<0.001**
Rich	8.72	6.74, 11.3	**<0.001**	8.84	6.77, 11.5	**<0.001**	11.7	10.1, 13.7	**<0.001**	9.35	8.31, 10.5	**<0.001**
**Sex of the household head**												
Male (ref.)	—	—		—	—		—	—		—	—	
Female	1.46	1.24, 1.73	**<0.001**	1.41	1.21, 1.65	**<0.001**	1.29	1.15, 1.45	**<0.001**	0.99	0.89, 1.09	0.78
**Age of the household head**												
<35 (ref.)	—	—		—	—		—	—		—	—	
35-54	1.47	1.29, 1.69	**<0.001**	1.71	1.51, 1.93	**<0.001**	1.45	1.30, 1.61	**<0.001**	1.26	1.15, 1.38	**<0.001**
54+	1.94	1.65, 2.30	**<0.001**	2.13	1.77, 2.58	**<0.001**	1.67	1.47, 1.90	**<0.001**	1.41	1.27, 1.55	**<0.001**
**Education of the household head**												
No education (ref.)	—	—		—	—		—	—		—	—	
Primary	1.56	1.31, 1.87	**<0.001**	1.61	1.38, 1.88	**<0.001**	1.37	1.21, 1.54	**<0.001**	1.25	1.14, 1.36	**<0.001**
Secondary	2.99	2.53, 3.54	**<0.001**	2.85	2.44, 3.33	**<0.001**	1.96	1.71, 2.25	**<0.001**	1.66	1.52, 1.82	**<0.001**
Higher	7.10	5.80, 8.69	**<0.001**	7.71	6.26, 9.49	**<0.001**	4.26	3.63, 5.00	**<0.001**	3.44	2.99, 3.96	**<0.001**
**Family size**												
1-4 (ref.)	—	—		—	—		—	—		—	—	
4+	1.18	1.06, 1.32	**0.003**	1.28	1.12, 1.46	**<0.001**	1.17	1.07, 1.29	**<0.001**	1.10	1.02, 1.19	**0.009**
**Mass media accessibility**												
With access (ref.)	—	—		—	—		—	—		—	—	
Without access	0.70	0.60, 0.81	**<0.001**	0.58	0.49, 0.69	**<0.001**	0.70	0.63, 0.78	**<0.001**	0.75	0.69, 0.81	**<0.001**
**Owning mobile phone**												
Yes (ref.)	—	—		—	—		—	—		—	—	
No	0.73	0.57, 0.94	**0.013**	0.52	0.38, 0.70	**<0.001**	0.57	0.43, 0.75	**<0.001**	0.49	0.38, 0.65	**<0.001**

AOR = Adjusted Odds Ratio, CI = Confidence Interval

Household wealth status was significantly associated with improved WASH access. Compared to the poor households, the rich households were associated with 8.72 times odds (AOR: 8.72 [95% CI: 6.74, 11.3]; p < 0.001) of accessing improved WASH facilities in 2011, 8.84 times odds (AOR: 8.84 [95% CI: 6.77, 11.5]; p < 0.001) in 2014, 11.7 times odds (AOR: 11.7 [95% CI: 10.1, 13.7]; p < 0.001) in 2017–18, and 9.35 times odds (AOR: 9.35 [95% CI: 8.31, 10.5]; p < 0.001) in 2022. The odds of improved WASH access were higher with higher levels of education. Compared to households where the head had no formal education, households where the head had higher education were significantly associated with higher odds across all survey years, with 7.10 times odds (AOR: 7.10 [95% CI: 5.80, 8.69]; p < 0.001) in 2011, 7.71 times odds (AOR: 7.71 [95% CI: 6.26, 9.49]; p < 0.001) in 2014, 4.26 times odds (AOR: 4.26 [95% CI: 3.63, 5.00]; p < 0.001) in 2017–18, and 3.44 times odds (AOR: 3.44 [95% CI: 2.99, 3.96]; p < 0.001) in 2022.

Household headed by female were associated with higher odds of improved WASH facilities compared to household headed by male, with 46% higher in 2011 (AOR: 1.46 [95% CI: 1.24, 1.73]; p < 0.001), 41% higher in 2014 (AOR: 1.41 [95% CI: 1.21, 1.65]; p < 0.001), and 29% higher in 2017–18 (AOR: 1.29 [95% CI: 1.15, 1.45]; p < 0.001). Household headed by older aged adult (54+) were associated with higher odds of improved WASH facilities compared to household headed by young age adult (<35), with 94% higher odds in 2011 (AOR: 1.94 [95% CI: 1.65, 2.30]; p < 0.001), 2.13 times odds in 2014 (AOR: 2.13 [95% CI: 1.77, 2.58]; p < 0.001), 67% higher odds in 2017–18 (AOR: 1.67 [95% CI: 1.47, 1.90]; p < 0.001), and 41% higher odds in 2022 (AOR: 1.41 [95% CI: 1.27, 1.55]; p < 0.001).

Family size was significantly associated with improved WASH access. Across all survey years, households with more than four members were associated with higher odds of accessing improved WASH facilities compared to one to four members, with 18% higher odds (AOR: 1.18 [95% CI: 1.06, 1.32]; p = 0.003) of accessing improved WASH facilities in 2011, 28% higher odds (AOR: 1.28 [95% CI: 1.12, 1.46]; p < 0.001) in 2014, 17% higher odds (AOR: 1.17 [95% CI: 1.07, 1.29]; p < 0.001) in 2017–18, and 10% higher odds (AOR: 1.10 [95% CI: 1.02, 1.19]; p = 0.009) in 2022. Media exposure was also associated with improved WASH access. Households without access to mass media were associated with lower odds of accessing improved WASH facilities compared to those with media access, with 30% lower odds (AOR: 0.70 [95% CI: 0.60, 0.81]; p < 0.001) in 2011, 42% lower odds (AOR: 0.58 [95% CI: 0.49, 0.69]; p < 0.001) in 2014, 30% lower odds (AOR: 0.70 [95% CI: 0.63, 0.78]; p < 0.001) in 2017–18, and 25% lower odds (AOR: 0.75 [95% CI: 0.69, 0.81]; p < 0.001) in 2022. Finally, compared to mobile phone-owning households, households that did not own mobile phones were associated with lower odds of having improved WASH facilities across all four survey years, with 27% lower odds (AOR: 0.73 [95% CI: 0.57, 0.94]; p = 0.013), 48% lower odds (AOR: 0.52 [95% CI: 0.38, 0.70]; p < 0.001), 43% lower odds (AOR: 0.57 [95% CI: 0.43, 0.75]; p < 0.001), and 51% lower odds (AOR: 0.49 [95% CI: 0.38, 0.65]; p < 0.001) in 2011, 2014, 2017–18, and 2022, respectively.

### Model validation

To access the multicollinearity in the fitted binary logistic regression models we performed the GVIF analysis (S2 and S3 Tables). Here all squared adjusted GVIF values below five, confirming the absence of multicollinearity in the fitted models.

### Classification tree analysis

**Fig 5:**
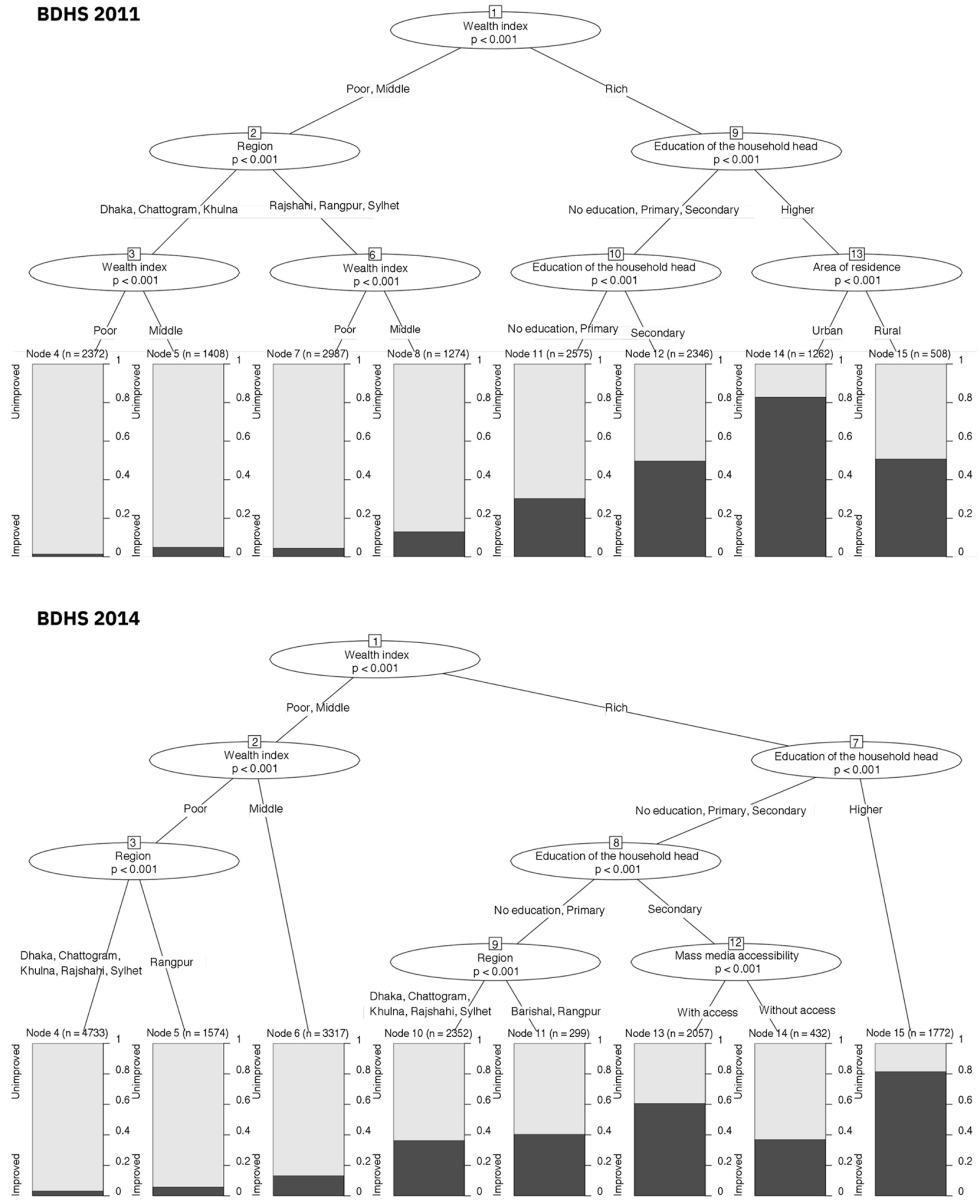
Classification tree illustrating the important predictors of WASH facilities in Bangladesh for 2011 and 2014.

**Fig 6:**
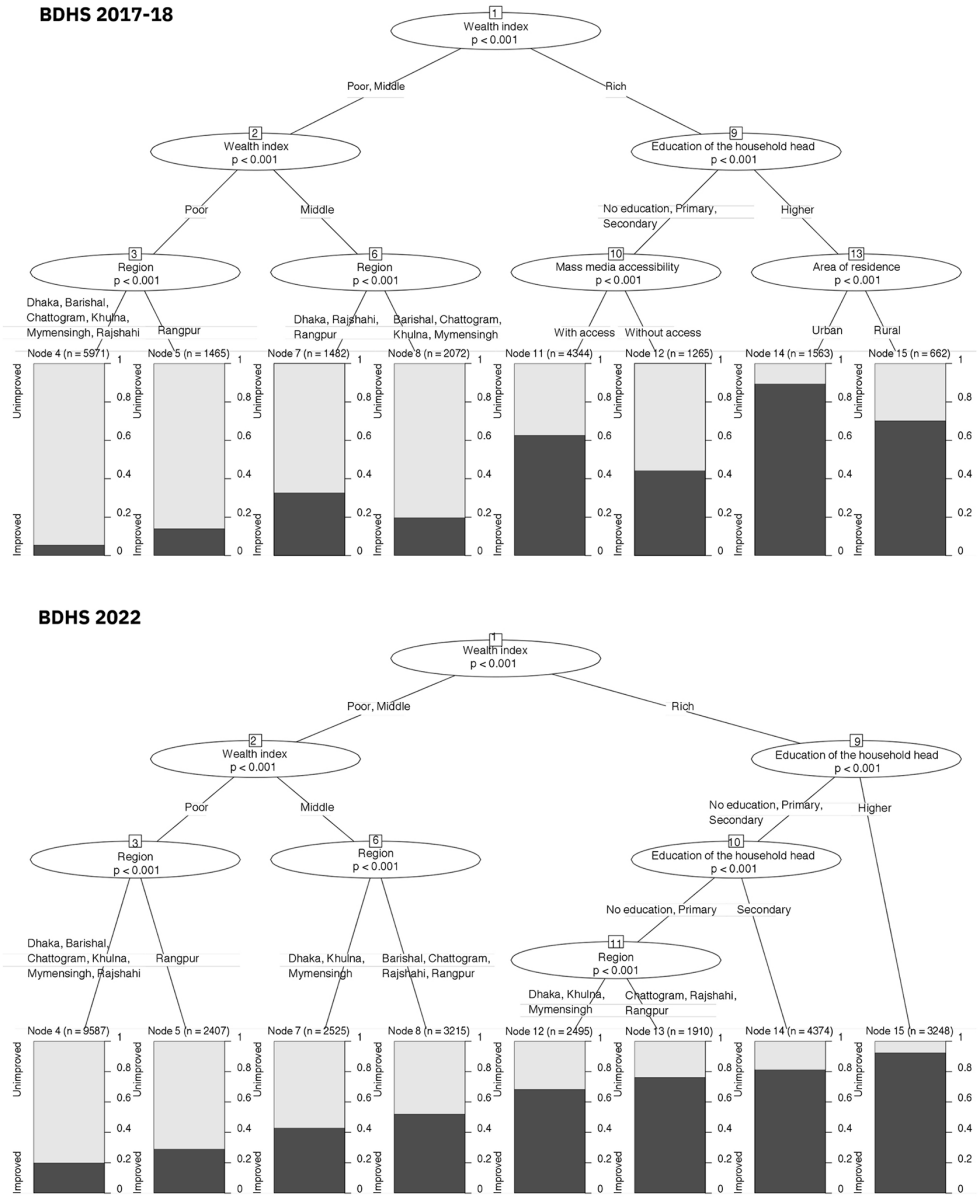
Classification tree illustrating the important predictors of WASH facilities in Bangladesh for 2017−18 and 2022.

The classification tree analysis (Figs 5 and 6) identified the wealth index as the most influential factor in determining WASH facility access, as it appeared at the top of the classification tree in all four survey years (2011, 2014, 2017–18, and 2022). Additionally, both region and the educational level of the household head remained crucial predictors throughout the period, consistent with earlier findings from regression models. Notably, the right-side nodes (representing rich households) showed the highest prevalence of improved WASH facilities, whereas the left-side nodes (corresponding to poorer and middle-income households) showed the lowest prevalence.

In 2011, node 4 showed that poor households from the Dhaka, Chattogram, and Khulna regions had the lowest coverage of improved WASH facilities (around 2%) across all subgroups. Similarly, nodes 5 and 7 identified additional vulnerable groups, including middle-income households from the Dhaka, Chattogram, and Khulna regions and poor households from the Rajshahi, Rangpur, and Sylhet regions, with slightly higher but still low coverage (around 5%).

In 2014, node 4 again showed that poor households from the Dhaka, Chattogram, Khulna, Rajshahi, and Sylhet regions had the lowest WASH coverage (around 3%). In addition, node 5 indicated that poor households from the Rangpur region also remained vulnerable, with similarly low coverage (around 6%).

In 2017–18, node 4 showed that poor households from the Dhaka, Barishal, Chattogram, Khulna, Mymensingh, and Rajshahi regions had the lowest coverage of improved WASH facilities (around 7%) across all subgroups. Similarly, node 5 indicated that poor households from the Rangpur region also remained vulnerable, with low coverage (around 14%).

In 2022, node 4 showed that poor households from the Dhaka, Barishal, Chattogram, Khulna, Mymensingh, and Rajshahi regions still had the lowest coverage of improved WASH facilities (around 20%) across all subgroups. Similarly, node 5 indicated that poor households from the Rangpur region also remained vulnerable, with relatively low coverage (around 28%).

## Discussion

Our study examined the key sociodemographic factors influencing WASH facilities in Bangladesh. Over the past decade, Bangladesh has made significant progress in improving WASH facilities across different socio-demographic groups of population. Notably, the primary factors influencing WASH access have remained largely unchanged over the past ten years (2011−2022). However, substantial disparities highlight the ongoing challenges. Despite governmental and non-governmental efforts to expand WASH coverage, key sociodemographic factors such as economic status, geographic region, family size, age and education level of the household head, access to mass media, and mobile phone ownership continue to play a significant role in determining access to these essential services. Notably, the observed improvements in hygiene facilities between 2017–18 and 2022 (16.76%) may be attributed to enhanced awareness and behavioral changes driven by the COVID-19 pandemic [45,46]. Despite these improvements, nearly half of the population still lacks access to adequate WASH services, particularly in rural (56%) and low-income communities (77%). These patterns suggest that, despite overall progress, economic constraints remain a central barrier to equitable WASH access, warranting closer examination of the role of household financial capacity.

Our findings indicate that financial constraints continue to be a major obstacle for low-income households. From both logistic regression and classification tree analyses household economic condition appeared as the most important predictor of improved WASH facilities. Previous study conducted in Sub-Saharan Africa [31], South Aisa [14], Bangladesh [15], and Pakistan [21] also found household economic condition significantly associated with WASH facilities. This disparity is primarily due to the financial capacity of wealthier households to invest in safe WASH infrastructure and their higher likelihood of residing in metropolitan areas with better access to improved services [47,48]. This pattern suggests that improvements in national WASH coverage may disproportionately benefit economically advantaged households, while poorer households continue to face affordability constraints and limited access to basic infrastructure. In the Bangladesh context, this may also reflect differences in service availability between urban and rural areas, as well as variations in the effectiveness of local-level implementation of WASH programs.

We found that family size was significantly associated with improved WASH access across all survey years. Evidence from studies in South Asia [14] and Bangladesh [15] also supports this conclusion. Further across all survey years age of the household head was significantly associated with improved WASH facilities. Household headed by older aged adult (54+) were associated with higher odds of improved WASH facilities compared to household headed by young age adult (<35). The older household heads are more likely to invest in and maintain better WASH facilities due to greater financial stability and awareness of hygiene practices [49,50]. This highlights the importance of considering demographic factors when formulating policies to enhance WASH access. These findings received additional support from studies conducted in Benin [51], Ethiopia [52], and South Asia [14]. These findings indicate that household-level demographic characteristics influence not only the capacity to invest in WASH facilities but also the prioritization of such investments. They further suggest that behavioral and life-cycle factors play a role in shaping WASH access beyond purely economic considerations.

Our findings revealed that education of household head was significantly associated with WASH facilities. Household with higher educated head had better access to WASH facilities (47.83% higher) compared to household head with no education. They are also more likely to be aware and prioritize investments in WASH infrastructure. Additionally, education is closely linked to economic stability [53], allowing educated individuals to afford better housing with modern WASH facilities. This assertion is further supported by research conducted in other LMICs [14,15,21,26,54]. This highlights that awareness and knowledge act as enabling factors that translate economic and infrastructural availability into actual WASH adoption, suggesting that improvements in service provision alone may not be sufficient without parallel investments in education and behavioral change.

Regional mapping indicates significant disparities in WASH coverage across different regions of Bangladesh. These findings are consistent with results from logistic regression analysis and classification tree analysis, which identify region as a key predictor of household-level WASH coverage. While regions such as Dhaka, Rajshahi, and Chattogram consistently demonstrate higher WASH coverage over the period, Barishal and Mymensingh have lagged behind in most survey years. These results align with recent studies in Bangladesh, which highlight that Barishal, Mymensingh, and Sylhet perform worse than other regions in terms of WASH access [15]. This may be due to their geographic location, as these areas are remote, riverine, and wetland-dominated [14]. Similar regional disparities have also been observed in other low- and middle-income countries (LMICs) globally [31,54,55]. These disparities likely reflect broader structural differences, including uneven infrastructure development, geographic accessibility, and variations in local governance and service delivery systems. In Bangladesh, such differences are particularly pronounced in flood-prone and remote regions, where environmental and logistical constraints limit the effectiveness of standard WASH interventions.

Our findings underscored the importance of accessing information in ensuring adequate WASH facilities. Access to information through media and mobile phones enhances awareness and understanding of the importance of improved WASH facilities, ultimately improving overall WASH facilities [56,57]. Study conducted in Tanzania [58], and South Asia [14] also found mass media accessibility and mobile phone owning status significantly associated with WASH facilities. This suggests that access to information serves as a critical pathway through which households become aware of, and adopt, improved WASH practices, reinforcing the importance of integrating communication strategies into WASH programs.

Taken together, these findings suggest that the persistence of disparities in WASH access is not solely driven by individual household characteristics but is also shaped by broader structural and system-level factors. While national programs have contributed to overall improvements, gaps in infrastructure, service delivery, and local-level implementation continue to limit equitable access. In addition, environmental challenges, such as flooding and water contamination, may further constrain progress in certain regions. These results indicate that achieving equitable WASH access in Bangladesh requires not only expanding coverage but also addressing the underlying structural constraints that influence how services are distributed and utilized.

The main strength of this study is the use of nationally representative data from 2011 to 2022, enabling an in-depth analysis of decade-long progress and challenges in WASH access across Bangladesh. To our knowledge, few studies have examined these patterns using multiple large-scale datasets across survey rounds in an integrated manner. However, certain limitations exist. Reliance on self-reported data may introduce recall or social desirability biases, affecting the accuracy of estimates. Additionally, interpretations of progress or challenges are based on overall percentage changes across sociodemographic factors, as household samples vary across surveys. The classification of WASH indicators in this study is based on the WHO–UNICEF Joint Monitoring Programme (JMP) “improved” definitions to ensure comparability across survey rounds; however, these do not directly correspond to SDG service-level classifications (e.g., basic or safely managed), and may therefore overestimate actual access to safe services, particularly in Bangladesh, where issues such as water quality (e.g., arsenic contamination) and accessibility constraints remain important.

### Policy implications

The findings of this study have important implications for WASH policy and implementation in Bangladesh, particularly in the context of achieving SDG 6 targets. First, the strong and persistent influence of economic status suggests the need for targeted financial interventions, such as subsidies, microfinance support, or cost-sharing mechanisms, to improve access to sanitation and hygiene facilities among low-income households. These efforts could be aligned with ongoing national programs led by the Local Government Division (LGD). Second, the identification of high-risk population subgroups through classification tree analysis provides an opportunity for more targeted and data-driven interventions. For example, poor households from the Dhaka, Barishal, Chattogram, Khulna, Mymensingh, and Rajshahi regions, should be prioritized in resource allocation and program design. Third, regional disparities highlight the importance of context-specific planning. In geographically challenging areas, such as flood-prone and riverine regions like Barishal and Mymensingh, investments in climate-resilient and location-adapted sanitation and hygiene infrastructure are essential. Strengthening coordination between national strategies and local-level NGOs can improve service delivery in these areas. Finally, the significant role of media exposure and mobile phone access suggests that behavioral change communication strategies should be further strengthened. Integrating WASH awareness campaigns into digital platforms and community-based outreach programs may enhance adoption, particularly among low-literacy and rural populations.

## Conclusion

This study examines decade-long changes in WASH coverage in Bangladesh, highlighting shifts in socio-demographic distribution, persistent regional disparities, and key factors influencing access. While overall progress is notable, substantial challenges remain, with a large proportion of the population still lacking adequate WASH services. Economic status emerges as the strongest determinant of improved access, followed by household characteristics such as family size, age, and education of the household head. Regional inequalities further emphasize the need for targeted interventions in underserved areas such as Barishal, Mymensingh, and Sylhet. In addition, access to information through media and mobile phones plays an important role in promoting WASH adoption. Achieving universal WASH coverage and SDG 6 targets will require targeted, context-specific strategies that address socio-economic inequalities, strengthen region-specific interventions, and enhance awareness and accessibility.

## Supporting information

S1 Table Average Annual Rate of Increase (AARI) of improved WASH components in Bangladesh, 2011–2022. (DOCX)

S2 Table GVIF for binary logistic regression model adjusted for demographic, socio-economic, and geographic factors with WASH facilities as outcome for BDHS 2011 and 2014. (DOCX)

S3 Table GVIF for binary logistic regression model adjusted for demographic, socio-economic, and geographic factors with WASH facilities as outcome for BDHS 2017−18 and 2022. (DOCX)
