# Supplementary material for: A decade of progress and challenges in Water, Sanitation and Hygiene (WASH) coverage in Bangladesh: Insights from Bangladesh demographic and health survey 2011–2022
Source: PLoS One. 2026 Jul 23;21(7):e0354480. doi: 10.1371/journal.pone.0354480 (PMC13395418; doi:10.1371/journal.pone.0354480)
Supplement: S1 Table — (DOCX) [file pone.0354480.s001.docx]

S1 Table: Average Annual Rate of Increase (AARI) of improved WASH components in Bangladesh, 2011–2022.

| **Indicator** | **BDHS 2011: Coverage (%)** | **AARI 2011-2014 (%)** | **BDHS 2014: Coverage (%)** | **AARI 2014-2017/18 (%)** | **BDHS 2017-18: Coverage (%)** | **AARI 2017/18-2022 (%)** | **BDHS 2022: Coverage (%)** | **AARI 2011-2022 (%)** |
| --- | --- | --- | --- | --- | --- | --- | --- | --- |
| **Improved drinking water facilities** | 98.90 | -0.20 | 98.32 | 0.03 | 98.43 | 0.18 | 99.14 | 0.02 |
| **Improved sanitation facilities** | 60.31 | 6.53 | 72.92 | -1.04 | 69.92 | 4.24 | 82.56 | 2.90 |
| **Improved hygiene facilities** | 24.97 | 4.71 | 28.67 | 7.80 | 38.71 | 9.41 | 55.47 | 7.53 |
| **Improved WASH facilities** | 22.13 | 6.06 | 26.40 | 6.47 | 33.93 | 10.27 | 50.17 | 7.72 |
