# Supplementary material for: A decade of progress and challenges in Water, Sanitation and Hygiene (WASH) coverage in Bangladesh: Insights from Bangladesh demographic and health survey 2011–2022
Source: PLoS One. 2026 Jul 23;21(7):e0354480. doi: 10.1371/journal.pone.0354480 (PMC13395418; doi:10.1371/journal.pone.0354480)
Supplement: S3 Table — (DOCX) [file pone.0354480.s003.docx]

S3 Table: GVIF for binary logistic regression model adjusted for demographic, socio-economic, and geographic factors with WASH facilities as outcome for BDHS 2017-18 and 2022.

|  | **BDHS 2017-18** | | | | **BDHS 2022** | | | |
| --- | --- | --- | --- | --- | --- | --- | --- | --- |
| **Variables** | **GVIF** | **Df** | **Adjusted GVIF** | **Squared adjusted GVIF** | **GVIF** | **Df** | **Adjusted GVIF** | **Squared adjusted GVIF** |
| **Area of residence** | 1.09 | 1 | 1.04 | 1.02 | 1.19 | 1 | 1.09 | 1.04 |
| **Region** | 1.34 | 7 | 1.02 | 1.01 | 1.38 | 7 | 1.02 | 1.01 |
| **Wealth index** | 1.36 | 2 | 1.08 | 1.04 | 1.21 | 2 | 1.05 | 1.02 |
| **Sex of the household head** | 1.16 | 1 | 1.08 | 1.04 | 1.16 | 1 | 1.08 | 1.04 |
| **Age of the household head** | 1.23 | 2 | 1.05 | 1.03 | 1.28 | 2 | 1.06 | 1.03 |
| **Education of the household head** | 1.26 | 3 | 1.04 | 1.02 | 1.36 | 3 | 1.05 | 1.03 |
| **Family size** | 1.15 | 1 | 1.07 | 1.04 | 1.13 | 1 | 1.06 | 1.03 |
| **Mass media accessibility** | 1.22 | 1 | 1.10 | 1.05 | 1.08 | 1 | 1.04 | 1.02 |
| **Owning mobile phone** | 1.11 | 1 | 1.05 | 1.03 | 1.09 | 1 | 1.05 | 1.02 |
