# Supplementary material for: A decade of progress and challenges in Water, Sanitation and Hygiene (WASH) coverage in Bangladesh: Insights from Bangladesh demographic and health survey 2011–2022
Source: PLoS One. 2026 Jul 23;21(7):e0354480. doi: 10.1371/journal.pone.0354480 (PMC13395418; doi:10.1371/journal.pone.0354480)
Supplement: Attachment — Submitted filename: Comment to authors_PlosOne.pdf [file pone.0354480.s004.pdf]

Abstract

1) Line 16-17: The first sentence is generic. I would expect at least one quantified global or Bangladesh-specific burden estimate to anchor importance. Also, write the full abbreviation of WASH the 1st time you use it.

2) Line 22-25: It is not clear how "improved" WASH, sanitation, and hygiene are defined, nor whether the WHO/UNICEF JMP definitions are strictly followed and consistent across all four BDHS rounds. This should be explicit.

3) Authors mentioned spatial mapping, survey-weighted logistic regression, GVIF, and classification trees. For an abstract, this is heavy on technical jargon; consider prioritising what is essential for understanding the findings.

4) Reporting "sanitation (22%), hygiene (30.5%), and overall WASH facilities (28%)" needs clarification: are these absolute percentage-point increases or relative percentage changes? Please properly state that it is the percent change from 2011 to 2022.

5) Line 41-42: The statement "primary factors influencing WASH access have remained largely unchanged" is interesting but not supported with numerical evidence here. It would help to name those factors explicitly.

6) The policy recommendations are sound but generic. Please provide more specific policy recommendations.

Introduction

7) The introduction should more clearly situate Bangladesh in the global WASH and SDG-6 landscape: How does its progress compare with regional neighbours or LMIC averages? At present, the text largely rehearses the importance of WASH but not Bangladesh's relative performance. Please properly mention Bangladesh's standing in achieving the SDG 6 goals.

Methods

8) Line 171-73: Please mention whether descriptive statistics are weighted frequency and percentages.

9) Line 173-77: The authors correctly mentioned the use of survey-weighted models. The methods should specify whether clustering and stratification were accounted for. Also, clarify whether analyses were done separately by survey round or as pooled data with year fixed effects.

10) Line 177-78: How was multicollinearity assessed using GVIF, and what decision rule (threshold) was applied?

11) Line 179-184: Please specify which outcome (overall WASH vs component) was modelled in the classification tree analysis. How you handled survey weights in tree building and how overfitting was addressed.

12) Line 185: For Spatial analysis, indicate whether you used smoothed estimates or raw weighted proportions. Clarify whether statistical tests for spatial clustering (like Moran's I) were performed or if maps are descriptive only.

Results

13) I don't understand why the authors provided the same information in Table 1 and Figure 3, and also in text lines 201-203. Please remove Table 1 and add the value labels in the figure.

14) As the main stated objective of this paper is to demonstrate decadal progress in WASH coverage, I strongly recommend that the authors add a table presenting the Average Annual Rate of Increase (AARI) for each WASH indicator. This should be shown both overall and stratified by key background characteristics. It will allow readers to see whether improvements have accelerated or stalled in specific subgroups.

15) Line 332-336: It does not add anything in result section. Please transfer the text in the methods and Table 4 as a supplementary table.

16) Line 341-350: The abstract mentioned that wealth is the most important split. This is unsurprising and already evident from regression. For the tree to justify inclusion, the Results should highlight novel high-risk combinations (like "poorest quintile + large family size + no media access in specific divisions"), and quantify how much of the remaining unserved population is concentrated in these nodes.

17) As an optional but valuable addition, I encourage the authors to consider incorporating formal equity metrics, such as the concentration index and the slope index of inequality (SII), to quantify wealth- and education-related gradients in WASH coverage over time.

Discussion

18) The Discussion should be restructured around a clear set of messages, like what magnitude of national progress has been achieved, by component, and to what extent have regional and wealth inequalities narrowed, persisted, or widened? What plausible policy or programmatic explanations exist? Please don't repeat the result in this section.

19) The Discussion currently seems to restate the statistical associations (wealth, education, media access, phone ownership) without deeply exploring mechanisms or referring to implementation experience in Bangladesh (for example, roles of DPHE, NGO programs, national sanitation campaigns). Readers will expect linkage between numbers and real-world systems.

20) Please provide a dedicated **Policy Implication** section before the conclusion. The recommended actions ("prioritize economically marginalised groups, subsidize WASH equipment, awareness campaigns") are appropriate but broad. Consider being more concrete, like which subgroups, what types of investments, and how might the classification tree-identified high-risk nodes be used operationally?
