# Supplementary material for: A decade of progress and challenges in Water, Sanitation and Hygiene (WASH) coverage in Bangladesh: Insights from Bangladesh demographic and health survey 2011–2022
Source: PLoS One. 2026 Jul 23;21(7):e0354480. doi: 10.1371/journal.pone.0354480 (PMC13395418; doi:10.1371/journal.pone.0354480)
Supplement: Attachment — Submitted filename: Response to Reviewers.docx [file pone.0354480.s006.docx]

5 March 2026

Rajib Chowdhury

Academic Editor

PLOS ONE

RE: PLOS ONE Decision: Revision required [PONE-D-25-64861] - [EMID:e9ab643b302a71bf]

Dear Editor,

Thank you for proving us the opportunity to revise the paper titled “A Decade of Progress and Challenges in Water, Sanitation and Hygiene (WASH) Coverage in Bangladesh: Insights from Bangladesh Demographic and Health Survey 2011-2022” for publication in *PLOS ONE*.

We have addressed the reviewers’ comments in our responses and incorporated related changes into the original manuscript through a revision. For convenience, the reviewers’ comments are highlighted in blue, and our responses are in black font.

The primary changes made in the revised version of the manuscript include:

1. Revised methodology section clarified WASH indicator definitions, analytical framework, and comparability across survey rounds.
2. Revised results section improved clarity of findings, distinguished percentage point changes, incorporated the Average Annual Rate of Increase (AARI), and aligned descriptive and regression interpretations.
3. Revised discussion section restructured to reduce repetition and strengthened interpretation by incorporating underlying mechanisms and policy-relevant insights. A dedicated Policy Implications subsection has also been added.

In addition to the above, all authors have read the revised manuscript. Some minor changes throughout the revised version of the manuscript are made, mostly to improve clarity of expressions and correct grammatical errors.

We would like to thank you and reviewers for all the valuable feedback and suggestions.

Sincere regards,

The authors.

**Title:** A Decade of Progress and Challenges in Water, Sanitation and Hygiene (WASH) Coverage in Bangladesh: Insights from Bangladesh Demographic and Health Survey 2011-2022

Manuscript ID: **PONE-D-25-64861**

# Journal Requirements:

1. Please ensure that your manuscript meets PLOS ONE's style requirements, including those for file naming. The PLOS ONE style templates can be found at
   [https://journals.plos.org/plosone/s/file?id=wjVg/PLOSOne_formatting_sample_main_body.pdf](https://track.editorialmanager.com/CL0/https:%2F%2Fjournals.plos.org%2Fplosone%2Fs%2Ffile%3Fid=wjVg%2FPLOSOne_formatting_sample_main_body.pdf/1/010f019cfef2d654-7d86d042-a2d4-40b7-9e14-43d7d0484909-000000/cNrAj0wsL9rScpNZHJwdGXsErFEcWxe1UVq9qL5-x-s=254) and [https://journals.plos.org/plosone/s/file?id=ba62/PLOSOne_formatting_sample_title_authors_affiliations.pdf](https://track.editorialmanager.com/CL0/https:%2F%2Fjournals.plos.org%2Fplosone%2Fs%2Ffile%3Fid=ba62%2FPLOSOne_formatting_sample_title_authors_affiliations.pdf/1/010f019cfef2d654-7d86d042-a2d4-40b7-9e14-43d7d0484909-000000/mB4TS3N8tfX8cEZp1uR-a4v1bGj27erx_UsrApPNxdo=254)

**Authors’ response**

Thank you for the suggestion. We have ensured that our manuscript meets PLOS ONE's style requirements.

2. Please In the online submission form, you indicated that the secondary datasets analyzed in this study are available upon request from the Demographic and Health Surveys (DHS) Program. Access to the data requires registration and approval through the

DHS website: https://dhsprogram.com/data/ available-datasets. The authors do not have the authority to share the data directly.

All PLOS journals now require all data underlying the findings described in their manuscript to be freely available to other researchers, either 1. In a public repository, 2. Within the manuscript itself, or 3. Uploaded as supplementary information.

This policy applies to all data except where public deposition would breach compliance with the protocol approved by your research ethics board. If your data cannot be made publicly available for ethical or legal reasons (e.g., public availability would compromise patient privacy), please explain your reasons on resubmission and your exemption request will be escalated for approval.

**Authors’ response**

Thank you. We have revised the Data Availability statement to comply with the journal’s requirements and updated it in the “Data Availability” field of the submission form via the “Edit Submission” option.

*“The data used in this study were obtained from the Demographic and Health Surveys (DHS) Program and constitute third-party data that are not owned by the authors. The Bangladesh Demographic and Health Survey (BDHS) datasets are available upon registration and approval through the DHS Program website (*[*https://dhsprogram.com/data/available-datasets.cfm*](https://dhsprogram.com/data/available-datasets.cfm)*). The authors obtained access to the data through the standard request and approval process of the DHS Program and did not receive any special privileges or preferential access that would not be available to other researchers.”*

3. Your ethics statement should only appear in the Methods section of your manuscript. If your ethics statement is written in any section besides the Methods, please delete it from any other section.

**Authors’ response**

Thank you for your comment. The ethics statement is already included under the “Ethical Considerations” subsection within the Methods section. We have ensured that it does not appear in any other section of the manuscript.

*“****Ethical Considerations***

*No ethical approval was required for this study as we utilized publicly available secondary data from BDHS, which had been already approved by institutional review boards (IRBs) at ICF and the Bangladesh Medical Research Council (BMRC). Additionally, written informed consent was obtained from participants before data collection, ensuring voluntary participation, confidentiality, and the right to withdraw at any time. The datasets were fully anonymized before access and are available upon request for research purposes through the DHS website (*[*https://dhsprogram.com/*](https://dhsprogram.com/)*).*

*”*

4. [We note that Figure 4 in your submission contain [map/satellite] images which may be copyrighted. All PLOS content is published under the Creative Commons Attribution License (CC BY 4.0), which means that the manuscript, images, and Supporting Information files will be freely available online, and any third party is permitted to access, download, copy, distribute, and use these materials in any way, even commercially, with proper attribution. For these reasons, we cannot publish previously copyrighted maps or satellite images created using proprietary data, such as Google software (Google Maps, Street View, and Earth). For more information, see our copyright guidelines: http://journals.plos.org/plosone/s/licenses-and-copyright.

   We require you to either (1) present written permission from the copyright holder to publish these figures specifically under the CC BY 4.0 license, or (2) remove the figures from your submission:

   a. You may seek permission from the original copyright holder of Figure 4 to publish the content specifically under the CC BY 4.0 license.

   We recommend that you contact the original copyright holder with the Content Permission Form (http://journals.plos.org/plosone/s/file?id=7c09/content-permission-form.pdf) and the following text:
   “I request permission for the open-access journal PLOS ONE to publish XXX under the Creative Commons Attribution License (CCAL) CC BY 4.0 (http://creativecommons.org/licenses/by/4.0/). Please be aware that this license allows unrestricted use and distribution, even commercially, by third parties. Please reply and provide explicit written permission to publish XXX under a CC BY license and complete the attached form.”

   Please upload the completed Content Permission Form or other proof of granted permissions as an "Other" file with your submission.

   In the figure caption of the copyrighted figure, please include the following text: “Reprinted from [ref] under a CC BY license, with permission from [name of publisher], original copyright [original copyright year].”

   b. If you are unable to obtain permission from the original copyright holder to publish these figures under the CC BY 4.0 license or if the copyright holder’s requirements are incompatible with the CC BY 4.0 license, please either i) remove the figure or ii) supply a replacement figure that complies with the CC BY 4.0 license. Please check copyright information on all replacement figures and update the figure caption with source information. If applicable, please specify in the figure caption text when a figure is similar but not identical to the original image and is therefore for illustrative purposes only.
   The following resources for replacing copyrighted map figures may be helpful:

   USGS National Map Viewer (public domain): http://viewer.nationalmap.gov/viewer/
   The Gateway to Astronaut Photography of Earth (public domain): http://eol.jsc.nasa.gov/sseop/clickmap/
   Maps at the CIA (public domain): https://www.cia.gov/library/publications/the-world-factbook/index.html and https://www.cia.gov/library/publications/cia-maps-publications/index.html
   NASA Earth Observatory (public domain): http://earthobservatory.nasa.gov/
   Landsat: http://landsat.visibleearth.nasa.gov/
   USGS EROS (Earth Resources Observatory and Science (EROS) Center) (public domain): http://eros.usgs.gov/#
   Natural Earth (public domain): http://www.naturalearthdata.com/](We note that Figure 4 in your submission contain [map/satellite] images which may be copyrighted. All PLOS content is published under the Creative Commons Attribution License (CC BY 4.0), which means that the manuscript, images, and Supporting Information files will be freely available online, and any third party is permitted to access, download, copy, distribute, and use these materials in any way, even commercially, with proper attribution. For these reasons, we cannot publish previously copyrighted maps or satellite images created using proprietary data, such as Google software (Google Maps, Street View, and Earth). For more information, see our copyright guidelines: http://journals.plos.org/plosone/s/licenses-and-copyright.We require you to either (1) present written permission from the copyright holder to publish these figures specifically under the CC BY 4.0 license, or (2) remove the figures from your submission:a. You may seek permission from the original copyright holder of Figure 4 to publish the content specifically under the CC BY 4.0 license.We recommend that you contact the original copyright holder with the Content Permission Form (http://journals.plos.org/plosone/s/file?id=7c09/content-permission-form.pdf) and the following text:\“I request permission for the open-access journal PLOS ONE to publish XXX under the Creative Commons Attribution License (CCAL) CC BY 4.0 (http://creativecommons.org/licenses/by/4.0/). Please be aware that this license allows unrestricted use and distribution, even commercially, by third parties. Please reply and provide explicit written permission to publish XXX under a CC BY license and complete the attached form.\”Please upload the completed Content Permission Form or other proof of granted permissions as an \"Other\" file with your submission.In the figure caption of the copyrighted figure, please include the following text: \“Reprinted from [ref] under a CC BY license, with permission from [name of publisher], original copyright [original copyright year].\”b. If you are unable to obtain permission from the original copyright holder to publish these figures under the CC BY 4.0 license or if the copyright holder’s requirements are incompatible with the CC BY 4.0 license, please either i) remove the figure or ii) supply a replacement figure that complies with the CC BY 4.0 license. Please check copyright information on all replacement figures and update the figure caption with source information. If applicable, please specify in the figure caption text when a figure is similar but not identical to the original image and is therefore for illustrative purposes only.The following resources for replacing copyrighted map figures may be helpful:USGS National Map Viewer (public domain): http://viewer.nationalmap.gov/viewer/The Gateway to Astronaut Photography of Earth (public domain): http://eol.jsc.nasa.gov/sseop/clickmap/Maps at the CIA (public domain): https://www.cia.gov/library/publications/the-world-factbook/index.html and https://www.cia.gov/library/publications/cia-maps-publications/index.htmlNASA Earth Observatory (public domain): http://earthobservatory.nasa.gov/Landsat: http://landsat.visibleearth.nasa.gov/USGS EROS (Earth Resources Observatory and Science (EROS) Center) (public domain): http://eros.usgs.gov/#Natural Earth (public domain): http://www.naturalearthdata.com/)

**Authors’ response**

Apologies for any confusion. Figure 4 of our manuscript contain spatial mappings. This map is not copyrighted. This is original image created by us using the statistical software R. The R packages and shapefile used in this manuscript are listed on **page 8**; in the last paragraph of the Statistical analyses section. Besides, we did not use any R packages that source their maps from Google Maps. Instead, we utilized the administrative boundary shapefile of South Asian countries from an openly licensed database, the geoBoundaries Global Administrative Database (Runfola et al., 2020), which we have cited in the last paragraph of the Statistical analyses section.

**Reference:**

Runfola D, Anderson A, Baier H, Crittenden M, Dowker E, Fuhrig S, Goodman S, Grimsley G, Layko R, Melville G, Mulder M. geoBoundaries: A global database of political administrative boundaries. PloS one. 2020 Apr 24;15(4):e0231866.

5. [Please include captions for your Supporting Information files at the end of your manuscript, and update any in-text citations to match accordingly. Please see our Supporting Information guidelines for more information: http://journals.plos.org/plosone/s/supporting-information.](http://track.editorialmanager.com/CL0/http:%2F%2Fjournals.plos.org%2Fplosone%2Fs%2Fsupporting-information./1/010f019cfef2d654-7d86d042-a2d4-40b7-9e14-43d7d0484909-000000/QvJnyA7LYCaHrOKwW7QGU8_aWmmY_paXMpyGqZgOHvo=254)

**Authors’ response**

Thank you for raising the point. We have updated the manuscript accordingly and the following section has been added at the end of our manuscript (page 27 of the revised manuscript),

*“****Supporting information***

***S1 Table: Average Annual Rate of Increase (AARI) of improved WASH components in Bangladesh, 2011–2022.***

***S2 Table. GVIF for binary logistic regression model adjusted for demographic, socio-economic, and geographic factors with WASH facilities as outcome for BDHS 2011 and 2014.***

***S3 Table. GVIF for binary logistic regression model adjusted for demographic, socio-economic, and geographic factors with WASH facilities as outcome for BDHS 2017-18 and 2022.****”*

6. [If the reviewer comments include a recommendation to cite specific previously published works, please review and evaluate these publications to determine whether they are relevant and should be cited. There is no requirement to cite these works unless the editor has indicated otherwise.](http://track.editorialmanager.com/CL0/http:%2F%2Fjournals.plos.org%2Fplosone%2Fs%2Fsupporting-information./1/010f019cfef2d654-7d86d042-a2d4-40b7-9e14-43d7d0484909-000000/QvJnyA7LYCaHrOKwW7QGU8_aWmmY_paXMpyGqZgOHvo=254)

**Authors’ response**

Thank you for your comment. We have carefully reviewed the reviewer-suggested references and evaluated their relevance to our study. Relevant references have been incorporated into the revised manuscript where appropriate.

# Reviewer #1

**Reviewer’s comment**

Abstract

1. Line 16-17: The first sentence is generic. I would expect at least one quantified global or Bangladesh‑specific burden estimate to anchor importance. Also, write the full abbreviation of WASH the 1st time you use it.

**Authors’ response**

Thank you for your comment. We have revised the background section of the abstract including opening sentence to define WASH and include quantitative evidence.

*“****Background***
*Access to safe water, sanitation, and hygiene (WASH) remains a critical global public health challenge, with an estimated 2 billion people lacking safe drinking water and basic hygiene services. In Bangladesh, disparities in WASH access persist across regions and socioeconomic groups. This study assesses decade-long changes in WASH coverage, examines its distribution across socio-demographic groups, identifies key associated factors, and highlights underserved regions and population subgroups.”* (Page 1 of the revised Manuscript)

2. Line 22-25: It is not clear how “improved” WASH, sanitation, and hygiene are defined, nor whether the WHO/UNICEF JMP definitions are strictly followed and consistent across all four BDHS rounds. This should be explicit.

**Authors’ response**

Thank you for your comment. We have clarified in the Methods section of the abstract that WASH indicators were defined according to WHO–UNICEF Joint Monitoring Programme (JMP) 2017 guidelines and applied consistently across all survey rounds.

*“****Methods***
*Data from four rounds of the Bangladesh Demographic and Health Survey (BDHS) (2011–2022) were analyzed. WASH indicators were classified as improved or unimproved based on WHO–UNICEF Joint Monitoring Programme (JMP) 2017 guidelines and applied consistently across all survey rounds. Spatial mapping was used to assess regional disparities, while regression models identified factors associated with WASH facilities. A classification tree approach was used to explore interactions among predictors.”* (Page 1 of the revised Manuscript)

3. Authors mentioned spatial mapping, survey‑weighted logistic regression, GVIF, and classification trees. For an abstract, this is heavy on technical jargon; consider prioritising what is essential for understanding the findings.

**Authors’ response**

Thank you for your comment. We have simplified the Methods section of the abstract by reducing technical details and retaining only the key analytical approaches, while preserving clarity and essential methodological information.

*“****Methods***
*Data from four rounds of the Bangladesh Demographic and Health Survey (BDHS) (2011–2022) were analyzed. WASH indicators were classified as improved or unimproved based on WHO–UNICEF Joint Monitoring Programme (JMP) 2017 guidelines and applied consistently across all survey rounds. Spatial mapping was used to assess regional disparities, while regression models identified factors associated with WASH facilities. A classification tree approach was used to explore interactions among predictors.”* (Page 1 of the updated Manuscript)

4. Reporting “sanitation (22%), hygiene (30.5%), and overall WASH facilities (28%)” needs clarification: are these absolute percentage‑point increases or relative percentage changes? Please properly state that it is the percent change from 2011 to 2022.

**Authors’ response**

Thank you for your comment. We have clarified in the Results section of the abstract that the reported values represent absolute percentage point increases from 2011 to 2022.

*“**Over the decade (2011–2022), access to improved drinking water remained high (above 98%), with improvements in sanitation (22 percentage points), hygiene (30.5 percentage points), and overall WASH facilities (28 percentage points).”*

5. Line 41-42: The statement “primary factors influencing WASH access have remained largely unchanged” is interesting but not supported with numerical evidence here. It would help to name those factors explicitly.

**Authors’ response**

Thank you for your comment. We have revised the conclusion to explicitly identify the key factors influencing WASH access, including wealth status, education level, and regional disparities.

*“Key determinants of WASH access, including wealth, education, and regional disparities, remained largely unchanged over time.” (Page 1 of the updated Manuscript)*

6. The policy recommendations are sound but generic. Please provide more specific policy recommendations.

**Authors’ response**

Thank you for your comment. We have refined the policy recommendations in the conclusion section of the abstract to make them more specific and actionable, including targeted subsidies for socioeconomically disadvantaged households and region-specific interventions in underserved areas.

*“Achieving SDG 6 requires targeted subsidies for socioeconomically disadvantaged households, region-specific interventions in underserved areas such as Barishal and Mymensingh, and strengthened awareness programs.”* *(Page 1 of the updated Manuscript)*

Introduction

7. The introduction should more clearly situate Bangladesh in the global WASH and SDG‑6 landscape: How does its progress compare with regional neighbours or LMIC averages? At present, the text largely rehearses the importance of WASH but not Bangladesh’s relative performance. Please properly mention Bangladesh's standing in achieving the SDG 6 goals.

**Authors’ response**

Thank you for this valuable suggestion. We have revised the Introduction to better situate Bangladesh within the global WASH and SDG 6 context. Specifically, we added a new paragraph that compares Bangladesh’s progress with regional neighbours (Afghanistan, Nepal, and Pakistan) using recent comparative evidence, as well as with global trends reported by the WHO/UNICEF Joint Monitoring Programme (JMP). The following paragraph has been added to the revised manuscript (page 4):

“*In the context of SDG 6, Bangladesh shows differing levels of progress across WASH components compared with other LMICs, particularly in South Asia. A recent comparative analysis across Afghanistan, Bangladesh, Nepal, and Pakistan reported that overall WASH coverage in Bangladesh was 50.3%, which is lower than Nepal (75.3%) and Pakistan (59.5%), but higher than Afghanistan (33.5%)* *(14). Bangladesh has achieved very high access to improved drinking water, exceeding 98% coverage; however, this progress is not consistently reflected in sanitation and hygiene services, where coverage remains comparatively lower (14). Similar patterns have been reported in other LMICs, where progress in drinking water access has outpaced improvements in sanitation and hygiene services (8). According to the WHO/UNICEF Joint Monitoring Programme (JMP), access to safely managed sanitation services in Bangladesh remains below 40% and lower than global averages (8).*”

8. Methods:

Line 171-73: Please mention whether descriptive statistics are weighted frequency and percentages.

**Authors’ response**

Thank you for this comment. We have clarified that descriptive statistics were calculated using survey weights. The updated text can be found on page 7 of the revised manuscript,

*“Survey-weighted descriptive analyses were conducted to provide an overview of the data, with weighted frequencies and percentages used to describe the distribution of WASH facilities across demographic, socio-economic, and geographic factors.”*

9. Line 173-77: The authors correctly mentioned the use of survey‑weighted models. The methods should specify whether clustering and stratification were accounted for. Also, clarify whether analyses were done separately by survey round or as pooled data with year fixed effects.

**Authors’ response**

Thanks for your this important suggestion. We have revised the Statistical analyses subsection to clarify that all analyses accounted for the complex survey design by incorporating sampling weights, clustering at the primary sampling unit (PSU) level, and stratification using the *survey* package in R. We have also clarified that analyses were conducted separately for each survey round.

The updated text can be found on page 7 of the revised manuscript,

*“All analyses were conducted separately for each survey round, while accounting for the complex survey design through the incorporation of sampling weights, clustering at the primary sampling unit (PSU) level, and stratification using the survey package in R.”*

10. Line 177-78: How was multicollinearity assessed using GVIF, and what decision rule (threshold) was applied?

**Authors’ response**

Thank you for this helpful suggestion. We have clarified the multicollinearity assessment in the Statistical analyses subsection. The updated text can be found on page 7 of the revised manuscript,

“Multicollinearity among predictors in the fitted logistic regression models was assessed using Generalized Variance Inflation Factor (GVIF) scores. Because several predictors were categorical with multiple levels, squared adjusted GVIF values were used to allow comparison across variables with different degrees of freedom. Squared adjusted GVIF values below 5 were considered indicative of no serious multicollinearity (36).”

11. Line 179-184: Please specify which outcome (overall WASH vs component) was modelled in the classification tree analysis. How you handled survey weights in tree building and how overfitting was addressed.

**Authors’ response**

Thank you for this helpful comment. We have clarified the classification tree analysis in the Statistical analyses subsection. Specifically, we now explicitly state that the overall WASH facilities variable was used as the outcome. We have also described that survey weights were incorporated as case weights during tree construction and that overfitting was controlled using a predefined significance threshold (mincriterion) to limit tree growth.

The updated paragraph can be found on page 7-8 of the revised manuscript,

*“To capture complex predictor–outcome interactions, classification tree analysis was employed using the overall WASH facilities variable as the outcome. This method partitions the data through hypothesis testing at each node, selecting the predictor with the strongest association based on p-values. Survey weights were incorporated as case weights during tree construction to account for unequal probability of selection. The splitting process continued only when statistically significant relationships were identified, based on a predefined significance threshold (mincriterion), which helped control tree growth and reduce the risk of overfitting. Terminal nodes summarize the distribution of the outcome variable within each segment (37).”*

12. Line 185: For Spatial analysis, indicate whether you used smoothed estimates or raw weighted proportions. Clarify whether statistical tests for spatial clustering (like Moran’s I) were performed or if maps are descriptive only.

**Authors’ response**

Thank you for this suggestion. We have clarified in the Methods section that the geographical maps were based on raw survey-weighted proportions of WASH coverage at the regional level. We have also specified that no spatial smoothing techniques or formal statistical tests for spatial clustering (e.g., Moran’s I) were applied, and that the maps are presented for descriptive purposes only.

The updated text can be found on page 7 of the revised manuscript,

*“Geographical mapping was based on raw survey-weighted proportions of WASH coverage at the regional level to illustrate spatial variation and changes over time. No spatial smoothing techniques or formal statistical tests for spatial clustering (e.g., Moran’s I) were applied, and the maps are presented for descriptive purposes only.”*

Results

13. I don’t understand why the authors provided the same information in Table 1 and Figure 3, and also in text lines 201-203. Please remove Table 1 and add the value labels in the figure.

**Authors’ response**

Thank you for pointing this out. We have removed Table 1 to avoid duplication of information and retained Figure 3. The updated paragraph can be found on page 8 of the revised manuscript,

*“****Coverage of WASH facilities***

***Fig 3*** *illustrated the decade-long progress in* *improved drinking water, sanitation, hygiene, and overall WASH facilities in Bangladesh While access to drinking water remained consistently high at over 98%, sanitation facilities increased by 22%, from 60.31% in 2011 to 82.56% in 2022. However, improved hygiene facilities were significantly lower in 2011, with only 24.97% coverage. While this improved over the decade, 44.53% of households still lacked access to adequate hygiene facilities. Similarly, improved WASH facilities were limited in 2011 (22.13%) and showed progress over time; however, 49.83% of households still lacked access to improved WASH facilities in 2022.*

*(Insert* ***Fig 3*** *here)*

***Fig 3*** *Decade of progress in access to household-level improved WASH facilities* *in Bangladesh.”*

In addition, value labels have been added to the figure to improve readability.

14. As the main stated objective of this paper is to demonstrate decadal progress in WASH coverage, I strongly recommend that the authors add a table presenting the Average Annual Rate of Increase (AARI) for each WASH indicator. This should be shown both overall and stratified by key background characteristics. It will allow readers to see whether improvements have accelerated or stalled in specific subgroups.

**Authors’ response**

Thank you for this valuable suggestion. Following this recommendation, we have calculated the Average Annual Rate of Increase (AARI) to quantify changes in WASH coverage over time. AARI estimates are now presented in a new table (Table 2) for improved WASH coverage stratified by key demographic, socio-economic, and geographic characteristics. The following *Average Annual Rate of Increase (AARI)* subsection has been added to the results section in page 13-15 of the revised manuscript,

*“****Average Annual Rate of Increase (AARI)***

***Table 2*** *presents the Average Annual Rate of Increase (AARI) in improved WASH coverage across demographic, socio-economic, and geographic characteristics in Bangladesh between 2011 and 2022. Overall, WASH coverage increased across all population subgroups, although the rate of improvement varied considerably.*

*Substantial gains were observed among disadvantaged groups. Rural households experienced a higher annual increase (AARI = 11.20%) compared to urban households (3.53%). Similarly, the poorest households showed the fastest improvement (AARI = 19.93%), followed by middle-income households (16.48%), while the richest group exhibited comparatively slower growth (5.16%). Higher rates of increase were also observed among households without access to mass media (14.78%) and those without mobile phones (11.46%).*

*Regional variation in AARI was evident. Khulna (10.39%) and Barishal (10.19%) demonstrated relatively higher long-term growth, whereas Sylhet showed comparatively slower progress (5.82%). Temporal patterns further indicate fluctuations in growth across survey intervals, with some regions experiencing short-term declines or slower growth during specific periods.*

*Across education levels, households with no formal education exhibited the highest rate of improvement (14.86%), while those with higher education showed more modest increases (2.71%). Similar patterns were observed across age groups and sex of household head, where improvements were relatively consistent, with slightly higher rates among younger household heads.*

***Table 2*** *Average Annual Rate of Increase (AARI) of improved WASH facilities by demographic, socio-economic, and geographic factors in Bangladesh, 2011–2022.*

| ***Variables*** | ***BDHS 2011: Improved WASH (%)*** | ***AARI 2011-2014 (%)*** | ***BDHS 2014: Improved WASH (%)*** | ***AARI 2014-2017/18 (%)*** | ***BDHS 2017-18: Improved WASH (%)*** | ***AARI 2017/18-2022 (%)*** | ***BDHS 2022: Improved WASH (%)*** | ***AARI 2011-2022 (%)*** |
| --- | --- | --- | --- | --- | --- | --- | --- | --- |
| ***Area of residence*** |  |  |  |  |  |  |  |  |
| *Urban* | *44.74* | *1.41* | *46.66* | *3.62* | *53.79* | *5.07* | *65.56* | *3.53* |
| *Rural* | *13.71* | *9.92* | *18.21* | *9.22* | *25.91* | *14.21* | *44.09* | *11.20* |
| ***Region*** |  |  |  |  |  |  |  |  |
| *Dhaka* | *26.63* | *3.81* | *29.79* | *10.81* | *44.92* | *6.66* | *58.14* | *7.36* |
| *Barishal* | *11.53* | *17.80* | *18.85* | *-4.32* | *15.80* | *20.69* | *33.52* | *10.19* |
| *Chattogram* | *22.10* | *11.34* | *30.50* | *5.52* | *37.81* | *8.17* | *51.77* | *8.05* |
| *Khulna* | *16.78* | *8.39* | *21.37* | *7.33* | *28.36* | *15.11* | *49.80* | *10.39* |
| *Rajshahi* | *21.39* | *7.10* | *26.28* | *5.99* | *33.17* | *12.95* | *53.99* | *8.78* |
| *Rangpur* | *20.46* | *3.13* | *22.44* | *7.41* | *29.87* | *12.84* | *48.42* | *8.15* |
| *Sylhet* | *22.43* | *-4.89* | *19.30* | *11.55* | *29.88* | *8.74* | *41.78* | *5.82* |
| *Mymensingh* |  |  |  |  | *21.29* | *14.12* | *36.11* |  |
| ***Wealth index*** |  |  |  |  |  |  |  |  |
| *Poor* | *3.12* | *11.63* | *4.34* | *15.82* | *7.81* | *31.06* | *23.04* | *19.93* |
| *Middle* | *9.18* | *15.65* | *14.20* | *17.46* | *27.03* | *16.12* | *49.14* | *16.48* |
| *Rich* | *45.51* | *5.38* | *53.25* | *4.45* | *63.38* | *5.70* | *79.12* | *5.16* |
| ***Sex of the household head*** |  |  |  |  |  |  |  |  |
| *Male* | *22.06* | *6.18* | *26.41* | *6.59* | *34.09* | *10.36* | *50.57* | *7.83* |
| *Female* | *22.72* | *5.02* | *26.32* | *5.83* | *33.02* | *9.84* | *48.07* | *7.05* |
| ***Age of the household head*** |  |  |  |  |  |  |  |  |
| *<35* | *17.69* | *4.90* | *20.42* | *10.54* | *30.49* | *10.86* | *46.05* | *9.09* |
| *35-54* | *23.51* | *6.68* | *28.54* | *5.54* | *35.41* | *10.08* | *51.99* | *7.48* |
| *54+* | *23.84* | *5.82* | *28.25* | *4.91* | *34.22* | *9.91* | *49.93* | *6.95* |
| ***Education of the household head*** |  |  |  |  |  |  |  |  |
| *No education* | *7.59* | *10.17* | *10.15* | *16.41* | *18.64* | *16.93* | *34.85* | *14.86* |
| *Primary* | *14.87* | *7.78* | *18.62* | *8.88* | *26.17* | *13.51* | *43.44* | *10.24* |
| *Secondary* | *31.61* | *5.13* | *36.73* | *3.78* | *42.60* | *8.66* | *59.38* | *5.90* |
| *Higher* | *61.60* | *4.05* | *69.40* | *0.55* | *70.93* | *3.91* | *82.68* | *2.71* |
| ***Family size*** |  |  |  |  |  |  |  |  |
| *1-4* | *20.84* | *6.48* | *25.16* | *6.61* | *32.50* | *10.70* | *48.81* | *8.04* |
| *4+* | *23.41* | *5.91* | *27.81* | *6.49* | *35.76* | *9.90* | *52.17* | *7.56* |
| ***Mass media accessibility*** |  |  |  |  |  |  |  |  |
| *With access* | *38.44* | *5.94* | *45.70* | *3.06* | *51.56* | *5.52* | *63.93* | *4.73* |
| *Without access* | *7.92* | *7.76* | *9.91* | *14.88* | *17.26* | *20.24* | *36.08* | *14.78* |
| ***Owning mobile phone*** |  |  |  |  |  |  |  |  |
| *Yes* | *26.41* | *3.25* | *29.07* | *4.97* | *35.29* | *9.60* | *50.92* | *6.15* |
| *No* | *4.65* | *1.69* | *4.89* | *19.94* | *10.12* | *10.96* | *15.34* | *11.46* |

*Supplementary* ***S1 table*** *presents the AARI for individual WASH components.* *The overall AARI for improved WASH coverage was 7.72% over the study period. The improvements were most pronounced for hygiene (7.53%) and overall WASH (7.72%), followed by sanitation (2.90%), while access to improved drinking water remained nearly constant (0.02%) due to already high baseline coverage.”*

In addition, we have included AARI estimates for individual WASH components (drinking water, sanitation, hygiene, and overall WASH) in Supplementary Table S1.

*“S1 Table: Average Annual Rate of Increase (AARI) of improved WASH components in Bangladesh, 2011–2022.*

| ***Indicator*** | ***BDHS 2011: Coverage (%)*** | ***AARI 2011-2014 (%)*** | ***BDHS 2014: Coverage (%)*** | ***AARI 2014-2017/18 (%)*** | ***BDHS 2017-18: Coverage (%)*** | ***AARI 2017/18-2022 (%)*** | ***BDHS 2022: Coverage (%)*** | ***AARI 2011-2022 (%)*** |
| --- | --- | --- | --- | --- | --- | --- | --- | --- |
| ***Improved drinking water facilities*** | *98.90* | *-0.20* | *98.32* | *0.03* | *98.43* | *0.18* | *99.14* | *0.02* |
| ***Improved sanitation facilities*** | *60.31* | *6.53* | *72.92* | *-1.04* | *69.92* | *4.24* | *82.56* | *2.90* |
| ***Improved hygiene facilities*** | *24.97* | *4.71* | *28.67* | *7.80* | *38.71* | *9.41* | *55.47* | *7.53* |
| ***Improved WASH facilities*** | *22.13* | *6.06* | *26.40* | *6.47* | *33.93* | *10.27* | *50.17* | *7.72* |

*”*

Furthermore, the calculation and definition of AARI have been described in the Statistical Analyses subsection of the Methods to ensure methodological clarity. The updated text can be found on pages 7 of the revised manuscript,

*“The Average Annual Rate of Increase (AARI) was calculated to quantify changes in WASH coverage over time using survey-weighted proportions. AARI was defined as the geometric annual rate of change between survey periods, following established approaches in demographic and public health research (Setu et al., 2022). Both interval-specific (2011–2014, 2014–2017/18, and 2017/18–2022) and overall (2011–2022) estimates were computed to assess temporal trends in WASH coverage across demographic, socio-economic, and geographic factors.”*

15. Line 332-336: It does not add anything in result section. Please transfer the text in the methods and Table 4 as a supplementary table.

**Authors’ response**

Thank you for your suggestion. We have removed Table 4 from the main manuscript and relocated it to the supplementary materials (now presented as Supplementary **S2 table** and **S3 table**). The relevant methodological aspects have also been clearly described in the Methods section. However, we have retained a brief reference to these results in the Results section, as we believe it provides important context and supports the interpretation of the main findings. The updated text can be found on page 20 of the revised manuscript,

*“**To access the multicollinearity in the fitted binary logistic regression models we performed the GVIF analysis (****S2 table*** *and* ***S3 table****). Here all squared adjusted GVIF values below five, confirming the absence of multicollinearity in the fitted models.”*

16. Line 341-350: The abstract mentioned that wealth is the most important split. This is unsurprising and already evident from regression. For the tree to justify inclusion, the Results should highlight novel high‑risk combinations (like “poorest quintile + large family size + no media access in specific divisions”), and quantify how much of the remaining unserved population is concentrated in these nodes.

**Authors’ response**

Thank you for this valuable suggestion. We have revised the classification tree results to emphasize novel high-risk combinations of predictors, highlighting terminal nodes with the lowest WASH coverage that represent concentrated clusters of underserved populations across survey years. The revised Classification Tree Analysis subsection can be found on pages 20–21 of the revised manuscript,

*“****Classification tree analysis***

*The classification tree analysis (****Fig 5*** *and* ***Fig 6****) identified the wealth index as the most influential factor in determining WASH facility access, as it appeared at the top of the classification tree in all four survey years (2011, 2014, 2017–18, and 2022). Additionally, both region and the educational level of the household head remained crucial predictors throughout the period, consistent with earlier findings from regression models. Notably, the right-side nodes (representing rich households) showed the highest prevalence of improved WASH facilities, whereas the left-side nodes (corresponding to poorer and middle-income households) showed the lowest prevalence.*

*In 2011, node 4 showed that poor households from the Dhaka, Chattogram, and Khulna regions had the lowest coverage of improved WASH facilities (around 2%) across all subgroups. Similarly, nodes 5 and 7 identified additional vulnerable groups, including middle-income households from the Dhaka, Chattogram, and Khulna regions and poor households from the Rajshahi, Rangpur, and Sylhet regions, with slightly higher but still low coverage (around 5%).*

*In 2014, node 4 again showed that poor households from the Dhaka, Chattogram, Khulna, Rajshahi, and Sylhet regions had the lowest WASH coverage (around 3%). In addition, node 5 indicated that poor households from the Rangpur region also remained vulnerable, with similarly low coverage (around 6%).*

*In 2017–18, node 4 showed that poor households from the Dhaka, Barishal, Chattogram, Khulna, Mymensingh, and Rajshahi regions had the lowest coverage of improved WASH facilities (around 7%) across all subgroups. Similarly, node 5 indicated that poor households from the Rangpur region also remained vulnerable, with low coverage (around 14%).*

*In 2022, node 4 showed that poor households from the Dhaka, Barishal, Chattogram, Khulna, Mymensingh, and Rajshahi regions still had the lowest coverage of improved WASH facilities (around 20%) across all subgroups. Similarly, node 5 indicated that poor households from the Rangpur region also remained vulnerable, with relatively low coverage (around 28%).*

*(Insert* ***Fig 5*** *here)*

***Fig 5*** *Classification tree illustrating the important predictors of WASH facilities in Bangladesh for* ***2011*** *and* ***2014****.*

*(Insert* ***Fig 6*** *here)*

***Fig 6*** *Classification tree illustrating the important predictors of WASH facilities in Bangladesh for* ***2017-18*** *and* ***2022****.”*

17. As an optional but valuable addition, I encourage the authors to consider incorporating formal equity metrics, such as the concentration index and the slope index of inequality (SII), to quantify wealth‑ and education‑related gradients in WASH coverage over time.

**Authors’ response**

Thank you for this valuable suggestion. We agree that formal equity metrics such as the concentration index and slope index of inequality (SII) can provide complementary insights into wealth- and education-related disparities in WASH coverage. However, the objective of the current study was to assess the distribution of WASH coverage across socio-demographic groups, identify associated factors, and explore high-risk population subgroups. Therefore, incorporating additional equity indices was beyond the scope of the present study.

Discussion

18. The Discussion should be restructured around a clear set of messages, like what magnitude of national progress has been achieved, by component, and to what extent have regional and wealth inequalities narrowed, persisted, or widened? What plausible policy or programmatic explanations exist? Please don’t repeat the result in this section.
19. The Discussion currently seems to restate the statistical associations (wealth, education, media access, phone ownership) without deeply exploring mechanisms or referring to implementation experience in Bangladesh (for example, roles of DPHE, NGO programs, national sanitation campaigns). Readers will expect linkage between numbers and real‑world systems.

**Authors’ response (Response to Comments 18 and 19)**

Thank you for these valuable suggestions. We have revised the Discussion section to improve its structure and provide clearer, high-level interpretation of the findings. The revised Discussion now focuses on the overall magnitude of national progress while emphasizing the persistence of socio-economic and regional disparities. In addition, we have strengthened the interpretation by moving beyond the description of statistical associations and incorporating explanations of the underlying mechanisms. Specifically, we discuss how structural factors such as affordability constraints, urban–rural infrastructure gaps, service delivery limitations, governance differences, access to information, and climate-related vulnerabilities contribute to the persistence of inequalities in WASH access. The revised Discussion section can be found on pages 22–25 of the revised manuscript,

*“****Discussion***

*Our study examined the key sociodemographic factors influencing WASH facilities in Bangladesh. Over the past decade, Bangladesh has made significant progress in improving WASH facilities across different socio-demographic groups of population. Notably, the primary factors influencing WASH access have remained largely unchanged over the past ten years (2011-2022). However, substantial disparities highlight the ongoing challenges. Despite governmental and non-governmental efforts to expand WASH coverage, key sociodemographic factors such as economic status, geographic region, family size, age and education level of the household head, access to mass media, and mobile phone ownership continue to play a significant role in determining access to these essential services. Notably, the observed improvements in hygiene facilities between 2017–18 and 2022 (16.76%) may be attributed to enhanced awareness and behavioral changes driven by the COVID-19 pandemic (45,46). Despite these improvements, nearly half of the population still lacks access to adequate WASH services, particularly in rural (56%) and low-income communities (77%). These patterns suggest that, despite overall progress, economic constraints remain a central barrier to equitable WASH access, warranting closer examination of the role of household financial capacity.*

*Our findings indicate that financial constraints continue to be a major obstacle for low-income households. From both logistic regression and classification tree analyses household economic condition appeared as the most important predictor of improved WASH facilities. Previous study conducted in Sub-Saharan Africa (31), South Aisa (14), Bangladesh (15), and Pakistan (21) also found household economic condition significantly associated with WASH facilities. This disparity is primarily due to the financial capacity of wealthier households to invest in safe WASH infrastructure and their higher likelihood of residing in metropolitan areas with better access to improved services (47,48). This pattern suggests that improvements in national WASH coverage may disproportionately benefit economically advantaged households, while poorer households continue to face affordability constraints and limited access to basic infrastructure. In the Bangladesh context, this may also reflect differences in service availability between urban and rural areas, as well as variations in the effectiveness of local-level implementation of WASH programs.*

*We found that family size was significantly associated with improved WASH access across all survey years. Evidence from studies in South Asia (14) and Bangladesh (15) also supports this conclusion. Further across all survey years age of the household head was significantly associated with improved WASH facilities. Household headed by older aged adult (54+) were associated with higher odds of improved WASH facilities compared to household headed by young age adult (<35). The older household heads are more likely to invest in and maintain better WASH facilities due to greater financial stability and awareness of hygiene practices (49,50). This highlights the importance of considering demographic factors when formulating policies to enhance WASH access. These findings received additional support from studies conducted in Benin (51), Ethiopia (52), and South Asia (14). These findings indicate that household-level demographic characteristics influence not only the capacity to invest in WASH facilities but also the prioritization of such investments. They further suggest that behavioral and life-cycle factors play a role in shaping WASH access beyond purely economic considerations.*

*Our findings revealed that education of household head was significantly associated with WASH facilities. Household with higher educated head had better access to WASH facilities (47.83 % higher) compared to household head with no education. They are also more likely to be aware and prioritize investments in WASH infrastructure. Additionally, education is closely linked to economic stability (53), allowing educated individuals to afford better housing with modern WASH facilities. This assertion is further supported by research conducted in other LMICs (14,15,21,26,54). This highlights that awareness and knowledge act as enabling factors that translate economic and infrastructural availability into actual WASH adoption, suggesting that improvements in service provision alone may not be sufficient without parallel investments in education and behavioral change.*

*Regional mapping indicates significant disparities in WASH coverage across different regions of Bangladesh. These findings are consistent with results from logistic regression analysis and classification tree analysis, which identify region as a key predictor of household-level WASH coverage. While regions such as Dhaka, Rajshahi, and Chattogram consistently demonstrate higher WASH coverage over the period, Barishal and Mymensingh have lagged behind in most survey years. These results align with recent studies in Bangladesh, which highlight that Barishal, Mymensingh, and Sylhet perform worse than other regions in terms of WASH access (15). This may be due to their geographic location, as these areas are remote, riverine, and wetland-dominated (14). Similar regional disparities have also been observed in other low- and middle-income countries (LMICs) globally (31,54,55). These disparities likely reflect broader structural differences, including uneven infrastructure development, geographic accessibility, and variations in local governance and service delivery systems. In Bangladesh, such differences are particularly pronounced in flood-prone and remote regions, where environmental and logistical constraints limit the effectiveness of standard WASH interventions.*

*Our findings underscored the importance of accessing information in ensuring adequate WASH facilities. Access to information through media and mobile phones enhances awareness and understanding of the importance of improved WASH facilities, ultimately improving overall WASH facilities (56,57). Study conducted in Tanzania (58), and South Asia (14) also found mass media accessibility and mobile phone owning status significantly associated with WASH facilities. This suggests that access to information serves as a critical pathway through which households become aware of, and adopt, improved WASH practices, reinforcing the importance of integrating communication strategies into WASH programs.*

*Taken together, these findings suggest that the persistence of disparities in WASH access is not solely driven by individual household characteristics but is also shaped by broader structural and system-level factors. While national programs have contributed to overall improvements, gaps in infrastructure, service delivery, and local-level implementation continue to limit equitable access. In addition, environmental challenges, such as flooding and water contamination, may further constrain progress in certain regions. These results indicate that achieving equitable WASH access in Bangladesh requires not only expanding coverage but also addressing the underlying structural constraints that influence how services are distributed and utilized.*

*The main strength of this study is the use of nationally representative data from 2011 to 2022, enabling an in-depth analysis of decade-long progress and challenges in WASH access across Bangladesh. To our knowledge, few studies have examined these patterns using multiple large-scale datasets across survey rounds in an integrated manner. However, certain limitations exist. Reliance on self-reported data may introduce recall or social desirability biases, affecting the accuracy of estimates. Additionally, interpretations of progress or challenges are based on overall percentage changes across sociodemographic factors, as household samples vary across surveys. The classification of WASH indicators in this study is based on the WHO–UNICEF Joint Monitoring Programme (JMP) “improved” definitions to ensure comparability across survey rounds; however, these do not directly correspond to SDG service-level classifications (e.g., basic or safely managed), and may therefore overestimate actual access to safe services, particularly in Bangladesh, where issues such as water quality (e.g., arsenic contamination) and accessibility constraints remain important.”*

20. Please provide a dedicated Policy Implication section before the conclusion. The recommended actions (“prioritize economically marginalised groups, subsidize WASH equipment, awareness campaigns”) are appropriate but broad. Consider being more concrete, like which subgroups, what types of investments, and how might the classification tree-identified high‑risk nodes be used operationally?

**Authors’ response**

Thank you for this valuable suggestion. We have added a dedicated Policy Implications subsection at the end of the Discussion section to strengthen the practical relevance of the study. In this subsection, we provide more specific and actionable recommendations by linking our findings to context-specific strategies, identifying priority subgroups and regions, and outlining potential intervention approaches based on the classification tree analysis.

The newly added subsection can be found on pages 25 of the revised manuscript:

“***Policy Implications***

*The findings of this study have important implications for WASH policy and implementation in Bangladesh, particularly in the context of achieving SDG 6 targets. First, the strong and persistent influence of economic status suggests the need for targeted financial interventions, such as subsidies, microfinance support, or cost-sharing mechanisms, to improve access to sanitation and hygiene facilities among low-income households. These efforts could be aligned with ongoing national programs led by the Local Government Division (LGD). Second, the identification of high-risk population subgroups through classification tree analysis provides an opportunity for more targeted and data-driven interventions. For example, poor households from the Dhaka,* *Barishal, Chattogram, Khulna, Mymensingh, and Rajshahi regions, should be prioritized in resource allocation and program design. Third, regional disparities highlight the importance of context-specific planning. In geographically challenging areas, such as flood-prone and riverine regions like Barishal and Mymensingh, investments in climate-resilient and location-adapted sanitation and hygiene infrastructure are essential. Strengthening coordination between national strategies and local-level NGOs can improve service delivery in these areas. Finally, the significant role of media exposure and mobile phone access suggests that behavioral change communication strategies should be further strengthened. Integrating WASH awareness campaigns into digital platforms and community-based outreach programs may enhance adoption, particularly among low-literacy and rural populations.*”

# Reviewer #2

**Reviewer’s comment**

Major Comments

1. Novelty and Contribution

The manuscript claims (Lines 107–108, 428–429) that no study has comprehensively analyzed decade-long WASH progress using nationally representative data, or that this is the first such study. These statements are questionable. Several previous studies have used DHS, MICS, or similar datasets to examine WASH inequalities in Bangladesh.

Among others, the Bangladesh Health Watch publication “50 Years of Independence: Progress of Health Sector in Bangladesh” (University Press Limited, 2023) includes a dedicated chapter on WASH, where even 50 years of progress and challenges have been articulated.

The authors are encouraged to undertake a more comprehensive literature review, revisit these claims, and clearly articulate what additional knowledge this study contributes beyond the existing body of work.

**Authors’ response**

Thank you for this insightful suggestion. We have revised the relevant paragraphs in the Introduction and Conclusion to acknowledge previous studies and national reports, and have softened our earlier claims. We have also clarified the contribution of this study as providing an integrated analysis of temporal changes, socio-demographic distribution, associated factors, and high-risk population subgroups using nationally representative data.

The revised paragraphs in the Introduction can be found on page 4-5 of the revised manuscript,

*“**Research has identified significant demographic, socioeconomic, and regional disparities in WASH coverage across Bangladesh and other LMICs in South Asia (14,15,20–22). Many studies have examined factors associated with WASH and its association with various health outcomes at local and national levels (14,15,20,23–25). Key influencing factors include geographic location, economic constraints, household environment, income, ethnicity, education, infrastructure availability, and cultural beliefs (22,26–28). In addition, broader national assessments have attempted to document long-term progress and challenges in Bangladesh (29). However, there remains a lack of integrated analyses that simultaneously examine temporal changes, socio-demographic distribution, associated factors, and high-risk population subgroups within a unified framework using nationally representative data in Bangladesh.*

*To address this gap, this study aims to assess decade-long changes in WASH coverage, examine its distribution across socio-demographic groups, identify key factors associated with WASH access, and highlight underserved regions and population subgroups using four rounds of nationally representative Bangladesh Demographic and Health Surveys (BDHS) data from 2011 to 2022.”*

The revised paragraph in the discussion can be found on page 24-25 of the revised manuscript,

*“The main strength of this study is the use of nationally representative data from 2011 to 2022, enabling an in-depth analysis of decade-long progress and challenges in WASH access across Bangladesh. To our knowledge, few studies have examined these patterns using multiple large-scale datasets across survey rounds in an integrated manner. However, certain limitations exist. Reliance on self-reported data may introduce recall or social desirability biases, affecting the accuracy of estimates. Additionally, interpretations of progress or challenges are based on overall percentage changes across sociodemographic factors, as household samples vary across surveys.* *The classification of WASH indicators in this study is based on the WHO–UNICEF Joint Monitoring Programme (JMP) “improved” definitions to ensure comparability across survey rounds; however, these do not directly correspond to SDG service-level classifications (e.g., basic or safely managed), and may therefore overestimate actual access to safe services, particularly in Bangladesh, where issues such as water quality (e.g., arsenic contamination) and accessibility constraints remain important.”*

2. Definitional Ambiguity and Incomplete Analysis
   The manuscript relies on the term “improved WASH,” which was primarily used during the MDG era. In the SDG era (2015–2030), the monitoring framework has moved away from this terminology and uses service-level ladders. Continued use of “improved” without alignment to SDG definitions limits the usefulness of the findings for policy and programme purposes.
   a) Drinking Water
   Under the SDG framework, drinking water services are categorized as surface water, no service, unimproved, limited, basic, and safely managed. These indicators are central to SDG-6 monitoring, and their absence should be acknowledged as a limitation.
   BDHS variables (e.g., Q103, Q107, Q108) allow at least partial classification into “basic” and “safely managed” services. The manuscript reports that access to improved drinking water exceeds 98% in Bangladesh (Line 203). However, under the WHO/UNICEF JMP framework, an “improved source” does not necessarily imply safe or safely managed drinking water.
   In the Bangladesh context—where arsenic contamination and water quality remain significant concerns—this figure may overstate actual access to safe water. Furthermore, if collection time exceeds 30 minutes (Q103), the service cannot be classified even as “basic.” Therefore, such statements, if not properly qualified, may be misleading for policy interpretation. The authors should clarify this distinction or explicitly acknowledge it as a limitation.
   b) Sanitation
   Under the SDG framework, sanitation is classified as open defecation, unimproved, limited, basic, and safely managed. BDHS variables (Q110, Q111) capture shared sanitation, which is essential to distinguish between “limited” and “basic” services.
   With additional analysis of the same dataset, it is possible to derive SDG-relevant sanitation service levels. The current analysis stops short of this, which limits its policy relevance.
   c) Hygiene
   In the SDG framework, hygiene is classified as no facility, limited, and basic. The term “improved hygiene” is not used. Therefore, the use of this terminology in the manuscript is not consistent with current global monitoring standards and should be revised.

**Authors’ response**

Thank you for this important and insightful comment. We acknowledge that the SDG framework has shifted from the use of “improved” indicators toward service-level ladders for drinking water, sanitation, and hygiene. In the present study, we used the “improved WASH” classification based on the WHO–UNICEF Joint Monitoring Programme (JMP) definitions to ensure consistency across BDHS survey rounds and comparability over time. This approach is consistent with previous studies that have analyzed WASH disparities in Bangladesh and South Asia using large-scale survey data (Jitu & Masud, 2025; Yunus et al., 2021).

We further note that the analytical framework of this study focuses on WASH as a composite indicator, where access is considered improved only when all three components, water, sanitation, and hygiene, are classified as improved. Although this definition originates from the MDG era, it remains relevant for assessing overall gaps in WASH access, particularly in Bangladesh, where a substantial proportion of households still lack improved sanitation and hygiene facilities, which ultimately limits overall WASH access.

We agree that “improved” access does not necessarily imply “safely managed” services, especially in the context of Bangladesh, where issues such as water quality (e.g., arsenic contamination) and accessibility constraints remain important concerns. While BDHS variables allow partial classification into SDG service levels, a complete derivation of these categories would require additional assumptions and harmonization across survey rounds, which was beyond the scope of the current analysis.

Importantly, the objective of this study is not to generate SDG-compliant service-level indicators, but to examine the relational patterns and consistency of associations between socio-demographic factors and WASH access over time. In this context, maintaining a consistent definition of WASH across all survey rounds is essential to ensure comparability and to accurately assess temporal changes and underlying relationships

To address this concern, we have explicitly acknowledged this limitation in the manuscript and clarified that the reported estimates of “improved” WASH should not be interpreted as equivalent to SDG-defined service levels. We have added the following sentence at the limitation paragraph on page 25 of the revised manuscript for clarity:

*“The classification of WASH indicators in this study is based on the WHO–UNICEF Joint Monitoring Programme (JMP) “improved” definitions to ensure comparability across survey rounds; however, these do not directly correspond to SDG service-level classifications (e.g., basic or safely managed), and may therefore overestimate actual access to safe services, particularly in Bangladesh, where issues such as water quality (e.g., arsenic contamination) and accessibility constraints remain important.”*

***References***

*Jitu, M. H. I., & Masud, M. S. (2025). Demographic, socioeconomic and regional disparities in the coverage of water, sanitation and hygiene facilities in four South Asian Countries. https://doi.org/10.1371/journal.pone.0319754*

*Yunus, F. M., Ahmed, M. S., Islam, M. I., Das, M. C., & Khan, A. (2021). Mapping and situation analysis of basic WASH facilities at households in Bangladesh: Evidence from a nationally representative survey. In PLoS ONE (Vol. 16, Number 11 November). https://doi.org/10.1371/journal.pone.0259635*

Minor Comments

3. The descriptive results (Lines 250–251) suggest no notable differences in WASH coverage between male- and female-headed households. However, the regression results (Lines 304–305) indicate significantly higher odds of improved WASH access among female-headed households. The authors should clarify this apparent discrepancy and explain how adjustment for socioeconomic and demographic factors influences the observed relationship.

**Authors’ response**

Thank you for your observation. We agree that the descriptive analysis shows minimal differences in WASH coverage between male- and female-headed households across all survey years. In 2011, the proportion of households with improved WASH facilities was 22.06% for male-headed households and 22.72% for female-headed households, a difference of less than 1 percentage point, while the adjusted analysis showed significantly higher odds for female-headed households (AOR: 1.46 [95% CI: 1.24, 1.73]; p < 0.001). In 2014, the corresponding percentages were 26.41% and 26.32%, indicating virtually no difference, yet the adjusted model again showed significantly higher odds (AOR: 1.41 [95% CI: 1.21, 1.65]; p < 0.001). In 2017–18, the descriptive proportions were 34.09% and 33.02%, reflecting a small difference of about 1 percentage point, while the adjusted analysis remained statistically significant (AOR: 1.29 [95% CI: 1.15, 1.45]; p < 0.001). In 2022, the descriptive proportions were 50.57% and 48.07%, showing a modest difference of around 2 percentage points, and the adjusted association was not statistically significant (AOR: 0.99 [95% CI: 0.89, 1.09]; p = 0.78).

Overall, these findings indicate that unadjusted descriptive percentages show minimal differences between male- and female-headed households, whereas regression results reflect adjusted associations after accounting for socioeconomic and demographic covariates. This pattern is consistent with the expected behavior of multivariable models, where confounding factors such as wealth, education, and regional distribution influence the relationship and become evident only after adjustment. It also highlights the limitation of relying solely on unadjusted descriptive comparisons for interpreting associations at the socio-demographic level.

4. The manuscript applies classification tree analysis in addition to logistic regression models. However, the tree analysis appears to largely replicate the regression findings, particularly the dominant role of wealth in predicting WASH access. The authors should clarify the added analytical value of this approach or consider simplifying the analysis.

**Authors’ response**

Thank you for this valuable observation. We have revised the classification tree results to better highlight their added analytical value beyond the regression models. Specifically, the revised analysis emphasizes high-risk combinations of predictors by focusing on terminal nodes with the lowest WASH coverage, which represent clusters of underserved populations across survey years. This approach provides additional insights into how multiple socio-demographic factors interact to shape WASH access and identify vulnerable subgroups, rather than only identifying individual significant predictors as in the regression models. The revised Classification Tree Analysis subsection can be found on pages 20–21 of the revised manuscript,

*“****Classification tree analysis***

*The classification tree analysis (****Fig 5*** *and* ***Fig 6****) identified the wealth index as the most influential factor in determining WASH facility access, as it appeared at the top of the classification tree in all four survey years (2011, 2014, 2017–18, and 2022). Additionally, both region and the educational level of the household head remained crucial predictors throughout the period, consistent with earlier findings from regression models. Notably, the right-side nodes (representing rich households) showed the highest prevalence of improved WASH facilities, whereas the left-side nodes (corresponding to poorer and middle-income households) showed the lowest prevalence.*

*In 2011, node 4 showed that poor households from the Dhaka, Chattogram, and Khulna regions had the lowest coverage of improved WASH facilities (around 2%) across all subgroups. Similarly, nodes 5 and 7 identified additional vulnerable groups, including middle-income households from the Dhaka, Chattogram, and Khulna regions and poor households from the Rajshahi, Rangpur, and Sylhet regions, with slightly higher but still low coverage (around 5%).*

*In 2014, node 4 again showed that poor households from the Dhaka, Chattogram, Khulna, Rajshahi, and Sylhet regions had the lowest WASH coverage (around 3%). In addition, node 5 indicated that poor households from the Rangpur region also remained vulnerable, with similarly low coverage (around 6%).*

*In 2017–18, node 4 showed that poor households from the Dhaka, Barishal, Chattogram, Khulna, Mymensingh, and Rajshahi regions had the lowest coverage of improved WASH facilities (around 7%) across all subgroups. Similarly, node 5 indicated that poor households from the Rangpur region also remained vulnerable, with low coverage (around 14%).*

*In 2022, node 4 showed that poor households from the Dhaka, Barishal, Chattogram, Khulna, Mymensingh, and Rajshahi regions still had the lowest coverage of improved WASH facilities (around 20%) across all subgroups. Similarly, node 5 indicated that poor households from the Rangpur region also remained vulnerable, with relatively low coverage (around 28%).*

*(Insert* ***Fig 5*** *here)*

***Fig 5*** *Classification tree illustrating the important predictors of WASH facilities in Bangladesh for* ***2011*** *and* ***2014****.*

*(Insert* ***Fig 6*** *here)*

***Fig 6*** *Classification tree illustrating the important predictors of WASH facilities in Bangladesh for* ***2017-18*** *and* ***2022****.”*

5. Interpretation of Findings
   The discussion section largely reiterates statistical findings rather than providing deeper interpretation. For example, the strong association between wealth and WASH access is well established in the literature. The authors are encouraged to go beyond description and discuss why such inequalities persist despite improvements in coverage. Relevant factors may include amongst others are urban–rural infrastructure gaps, affordability constraints, governance, communication, access to TV and mobile phone, service delivery issues, and climate-related vulnerabilities.

**Authors’ response**

Thank you for this insightful comment. We agree that deeper interpretation of the findings is essential to strengthen the discussion. In response, we have revised the Discussion section to move beyond the description of statistical associations and provide a more comprehensive interpretation of the underlying mechanisms driving WASH disparities. Specifically, we now discuss how structural factors such as affordability constraints, urban–rural infrastructure gaps, service delivery limitations, governance differences, access to information, and climate-related vulnerabilities contribute to the persistence of inequalities despite overall improvements in coverage. We have incorporated these interpretative insights across the Discussion section to better contextualize the findings within the broader Bangladesh setting. The revised Discussion section can be found on pages 22–25 of the revised manuscript,

*“****Discussion***

*Our study examined the key sociodemographic factors influencing WASH facilities in Bangladesh. Over the past decade, Bangladesh has made significant progress in improving WASH facilities across different socio-demographic groups of population. Notably, the primary factors influencing WASH access have remained largely unchanged over the past ten years (2011-2022). However, substantial disparities highlight the ongoing challenges. Despite governmental and non-governmental efforts to expand WASH coverage, key sociodemographic factors such as economic status, geographic region, family size, age and education level of the household head, access to mass media, and mobile phone ownership continue to play a significant role in determining access to these essential services. Notably, the observed improvements in hygiene facilities between 2017–18 and 2022 (16.76%) may be attributed to enhanced awareness and behavioral changes driven by the COVID-19 pandemic (45,46). Despite these improvements, nearly half of the population still lacks access to adequate WASH services, particularly in rural (56%) and low-income communities (77%). These patterns suggest that, despite overall progress, economic constraints remain a central barrier to equitable WASH access, warranting closer examination of the role of household financial capacity.*

*Our findings indicate that financial constraints continue to be a major obstacle for low-income households. From both logistic regression and classification tree analyses household economic condition appeared as the most important predictor of improved WASH facilities. Previous study conducted in Sub-Saharan Africa (31), South Aisa (14), Bangladesh (15), and Pakistan (21) also found household economic condition significantly associated with WASH facilities. This disparity is primarily due to the financial capacity of wealthier households to invest in safe WASH infrastructure and their higher likelihood of residing in metropolitan areas with better access to improved services (47,48). This pattern suggests that improvements in national WASH coverage may disproportionately benefit economically advantaged households, while poorer households continue to face affordability constraints and limited access to basic infrastructure. In the Bangladesh context, this may also reflect differences in service availability between urban and rural areas, as well as variations in the effectiveness of local-level implementation of WASH programs.*

*We found that family size was significantly associated with improved WASH access across all survey years. Evidence from studies in South Asia (14) and Bangladesh (15) also supports this conclusion. Further across all survey years age of the household head was significantly associated with improved WASH facilities. Household headed by older aged adult (54+) were associated with higher odds of improved WASH facilities compared to household headed by young age adult (<35). The older household heads are more likely to invest in and maintain better WASH facilities due to greater financial stability and awareness of hygiene practices (49,50). This highlights the importance of considering demographic factors when formulating policies to enhance WASH access. These findings received additional support from studies conducted in Benin (51), Ethiopia (52), and South Asia (14). These findings indicate that household-level demographic characteristics influence not only the capacity to invest in WASH facilities but also the prioritization of such investments. They further suggest that behavioral and life-cycle factors play a role in shaping WASH access beyond purely economic considerations.*

*Our findings revealed that education of household head was significantly associated with WASH facilities. Household with higher educated head had better access to WASH facilities (47.83 % higher) compared to household head with no education. They are also more likely to be aware and prioritize investments in WASH infrastructure. Additionally, education is closely linked to economic stability (53), allowing educated individuals to afford better housing with modern WASH facilities. This assertion is further supported by research conducted in other LMICs (14,15,21,26,54). This highlights that awareness and knowledge act as enabling factors that translate economic and infrastructural availability into actual WASH adoption, suggesting that improvements in service provision alone may not be sufficient without parallel investments in education and behavioral change.*

*Regional mapping indicates significant disparities in WASH coverage across different regions of Bangladesh. These findings are consistent with results from logistic regression analysis and classification tree analysis, which identify region as a key predictor of household-level WASH coverage. While regions such as Dhaka, Rajshahi, and Chattogram consistently demonstrate higher WASH coverage over the period, Barishal and Mymensingh have lagged behind in most survey years. These results align with recent studies in Bangladesh, which highlight that Barishal, Mymensingh, and Sylhet perform worse than other regions in terms of WASH access (15). This may be due to their geographic location, as these areas are remote, riverine, and wetland-dominated (14). Similar regional disparities have also been observed in other low- and middle-income countries (LMICs) globally (31,54,55). These disparities likely reflect broader structural differences, including uneven infrastructure development, geographic accessibility, and variations in local governance and service delivery systems. In Bangladesh, such differences are particularly pronounced in flood-prone and remote regions, where environmental and logistical constraints limit the effectiveness of standard WASH interventions.*

*Our findings underscored the importance of accessing information in ensuring adequate WASH facilities. Access to information through media and mobile phones enhances awareness and understanding of the importance of improved WASH facilities, ultimately improving overall WASH facilities (56,57). Study conducted in Tanzania (58), and South Asia (14) also found mass media accessibility and mobile phone owning status significantly associated with WASH facilities. This suggests that access to information serves as a critical pathway through which households become aware of, and adopt, improved WASH practices, reinforcing the importance of integrating communication strategies into WASH programs.*

*Taken together, these findings suggest that the persistence of disparities in WASH access is not solely driven by individual household characteristics but is also shaped by broader structural and system-level factors. While national programs have contributed to overall improvements, gaps in infrastructure, service delivery, and local-level implementation continue to limit equitable access. In addition, environmental challenges, such as flooding and water contamination, may further constrain progress in certain regions. These results indicate that achieving equitable WASH access in Bangladesh requires not only expanding coverage but also addressing the underlying structural constraints that influence how services are distributed and utilized.*

*The main strength of this study is the use of nationally representative data from 2011 to 2022, enabling an in-depth analysis of decade-long progress and challenges in WASH access across Bangladesh. To our knowledge, few studies have examined these patterns using multiple large-scale datasets across survey rounds in an integrated manner. However, certain limitations exist. Reliance on self-reported data may introduce recall or social desirability biases, affecting the accuracy of estimates. Additionally, interpretations of progress or challenges are based on overall percentage changes across sociodemographic factors, as household samples vary across surveys. The classification of WASH indicators in this study is based on the WHO–UNICEF Joint Monitoring Programme (JMP) “improved” definitions to ensure comparability across survey rounds; however, these do not directly correspond to SDG service-level classifications (e.g., basic or safely managed), and may therefore overestimate actual access to safe services, particularly in Bangladesh, where issues such as water quality (e.g., arsenic contamination) and accessibility constraints remain important.”*

6. Policy Implications
   The policy recommendations are currently quite general. The manuscript would benefit from stronger linkage to specific national policies, sector strategies, or financing mechanisms in Bangladesh related to WASH and SDG implementation. This would enhance the practical relevance of the study.

**Authors’ response**

Thank you for this valuable suggestion. We have added a dedicated Policy Implications subsection at the end of the Discussion section to strengthen the practical relevance of the study. In this subsection, we provide more specific and actionable recommendations by linking our findings to context-specific strategies, identifying priority subgroups and regions, and outlining potential intervention approaches based on the classification tree analysis.

The newly added subsection can be found on pages 25 of the revised manuscript:

“***Policy Implications***

*The findings of this study have important implications for WASH policy and implementation in Bangladesh, particularly in the context of achieving SDG 6 targets. First, the strong and persistent influence of economic status suggests the need for targeted financial interventions, such as subsidies, microfinance support, or cost-sharing mechanisms, to improve access to sanitation and hygiene facilities among low-income households. These efforts could be aligned with ongoing national programs led by the Local Government Division (LGD). Second, the identification of high-risk population subgroups through classification tree analysis provides an opportunity for more targeted and data-driven interventions. For example, poor households from the Dhaka, Barishal, Chattogram, Khulna, Mymensingh, and Rajshahi regions, should be prioritized in resource allocation and program design. Third, regional disparities highlight the importance of context-specific planning. In geographically challenging areas, such as flood-prone and riverine regions like Barishal and Mymensingh, investments in climate-resilient and location-adapted sanitation and hygiene infrastructure are essential. Strengthening coordination between national strategies and local-level NGOs can improve service delivery in these areas. Finally, the significant role of media exposure and mobile phone access suggests that behavioral change communication strategies should be further strengthened. Integrating WASH awareness campaigns into digital platforms and community-based outreach programs may enhance adoption, particularly among low-literacy and rural populations.*”
