# Supplementary material for: A decade of progress and challenges in Water, Sanitation and Hygiene (WASH) coverage in Bangladesh: Insights from Bangladesh demographic and health survey 2011–2022
Source: PLoS One. 2026 Jul 23;21(7):e0354480. doi: 10.1371/journal.pone.0354480 (PMC13395418; doi:10.1371/journal.pone.0354480)
Supplement: Attachment — Submitted filename: Response_to_Reviewers_auresp_2.docx [file pone.0354480.s007.docx]

22 June 2026

Rajib Chowdhury

Academic Editor

PLOS ONE

RE: PLOS ONE Decision: Revision required [PONE-D-25-64861R1] - [EMID:5b1a1b4a25a68fe2]

Dear Editor,

Thank you for providing us the opportunity to revise the paper titled “A Decade of Progress and Challenges in Water, Sanitation and Hygiene (WASH) Coverage in Bangladesh: Insights from Bangladesh Demographic and Health Survey 2011–2022” for publication in PLOS ONE.

We have addressed the reviewers’ comments in our responses. For convenience, the reviewers’ comments are highlighted in blue, and our responses are presented in black font.

No substantive changes were made to the manuscript in this revision. The reviewers’ comments were addressed through clarification in the response document. The only changes to the manuscript relate to minor updates in reference numbering resulting from the revision process.

We would like to thank the editor and reviewers for their valuable feedback and constructive suggestions.

Sincere regards,

The Authors

**Title:** A Decade of Progress and Challenges in Water, Sanitation and Hygiene (WASH) Coverage in Bangladesh: Insights from Bangladesh Demographic and Health Survey 2011-2022

Manuscript ID: **PONE-D-25-64861R1**

# Journal Requirements:

1. [If the reviewer comments include a recommendation to cite specific previously published works, please review and evaluate these publications to determine whether they are relevant and should be cited. There is no requirement to cite these works unless the editor has indicated otherwise.](http://track.editorialmanager.com/CL0/http:%2F%2Fjournals.plos.org%2Fplosone%2Fs%2Fsupporting-information./1/010f019cfef2d654-7d86d042-a2d4-40b7-9e14-43d7d0484909-000000/QvJnyA7LYCaHrOKwW7QGU8_aWmmY_paXMpyGqZgOHvo=254)

**Authors’ response**

Thank you for your comment. We have carefully reviewed the reviewer-suggested references and evaluated their relevance to our study. Relevant references have been incorporated into the revised manuscript where appropriate.

# Reviewer #1

**Reviewer’s comment**

1. The revision addresses my comments well overall. Good luck. For the classification tree, I would still encourage the authors to report, for the lowest‑coverage terminal nodes, the proportion of all households lacking improved WASH that fall into these nodes, to better quantify how concentrated unmet need is within the identified high‑risk subgroups.

**Authors’ response**

Thank you for your suggestion. We agree that quantifying the proportion of households lacking improved WASH that are concentrated within the lowest-coverage terminal nodes would provide additional insight into the distribution of unmet need. However, the primary objective of the classification tree analysis in this study was to identify high-risk population subgroups and the interactions among key predictors associated with WASH access, rather than to quantify the concentration of unmet need across population segments. We therefore retained the current presentation focused on vulnerable population profiles. We appreciate this suggestion and recognize it as a valuable direction for future research.

# Reviewer #3

**Reviewer’s comment**

1. This first concern had already been raised by previous reviewers but does not appear to have been adequately addressed. The terminology “improved” versus “unimproved” corresponds to the classification used under the Millennium Development Goals (MDGs), which remained in effect until 2015. Since the adoption of the Sustainable Development Goals (SDGs), water, sanitation, and hygiene (WASH) services have been classified into the following categories: safely managed, basic, limited, unimproved, and no service.

   Based on the definitions adopted by the authors, the water sources categorized as “improved” in this study more closely correspond to “limited” water services under the SDG framework. Indeed, SDG limited water services include sources such as piped water into a dwelling, yard, or plot, public standpipes, boreholes or tube wells, protected dug wells, protected springs, rainwater, tanker trucks, carts with tanks, and bottled water. However, this classification does not take into account the time required to collect water. In contrast, a basic water service requires not only the use of an improved source but also that the total round-trip time for collecting water does not exceed 30 minutes.

   Basic sanitation services refer to the use of improved sanitation facilities, including flush or pour-flush toilets connected to a sewer system, septic tank, or pit latrine, improved pit latrines with slabs, and composting toilets. However, this category includes an additional requirement: the facility must not be shared with other households. By contrast, limited sanitation services refer to the same types of improved facilities when they are shared by two or more households. Therefore, the distinction between “basic” and “limited” sanitation services is based not on the type of facility used, but rather on whether its use is exclusive or shared.

   Basic hygiene facilities are handwashing facilities with soap and water available on the household premises.

   Consequently, the study’s focus on limited water and sanitation services appears misaligned with the SDG programmatic targets. SDG 1 aims to end poverty in all its forms and includes Target 1.4, which calls for universal access to basic services, particularly for poor and vulnerable populations.

**Authors’ response**

Thank you for your comment. We agree that the SDG framework classifies water, sanitation, and hygiene services using service-level ladders (safely managed, basic, limited, unimproved, and no service), which differ from the traditional WHO–UNICEF JMP improved/unimproved classifications. We also acknowledge that the distinction between basic and limited services depends on additional criteria, such as collection time for drinking water and sharing status for sanitation facilities.

We would like to note that a similar concern was raised by a previous reviewer and, in response, we revised the manuscript to clarify the rationale for adopting the WHO–UNICEF JMP improved/unimproved classification framework. The primary objective of this study was not to generate official SDG service-level indicators or SDG monitoring estimates. Rather, as stated in the **Introduction (Page 5)**:

*“To address this gap, this study aims to assess decade-long changes in WASH coverage, examine its distribution across socio-demographic groups, identify key factors associated with WASH access, and highlight underserved regions and population subgroups using four rounds of nationally representative Bangladesh Demographic and Health Surveys (BDHS) data from 2011 to 2022.”*

To achieve this objective, it was essential to apply a consistent classification framework across all four BDHS survey rounds. Therefore, WASH indicators were classified according to the WHO–UNICEF JMP improved/unimproved definitions and applied uniformly throughout the study period. This approach enabled meaningful assessment of temporal changes, socio-demographic inequalities, associated factors, and high-risk population subgroups over the 2011–2022 period.

We have also explicitly acknowledged this limitation in the revised manuscript (**Discussion section, Page 25**), where we state:

*“The classification of WASH indicators in this study is based on the WHO–UNICEF Joint Monitoring Programme (JMP) ‘improved’ definitions to ensure comparability across survey rounds; however, these do not directly correspond to SDG service-level classifications (e.g., basic or safely managed), and may therefore overestimate actual access to safe services, particularly in Bangladesh, where issues such as water quality (e.g., arsenic contamination) and accessibility constraints remain important.”*

Therefore, while SDG service-level classifications are important for global monitoring and programmatic evaluation, the use of the JMP improved/unimproved framework was necessary to achieve the objectives of the present study and ensure methodological consistency and comparability across all BDHS survey rounds. Furthermore, similar nationally representative studies conducted in Bangladesh have adopted the WHO–UNICEF JMP improved/unimproved classification framework to assess WASH coverage, inequalities, and associated factors, supporting its continued utility for population-level trend and disparity analyses (Ahmed et al., 2021).

***Reference***

*Ahmed MS, Islam MI, Das MC, Khan A, Yunus FM. Mapping and situation analysis of basic WASH facilities at households in Bangladesh: Evidence from a nationally representative survey. PLoS ONE. 2021. doi:10.1371/journal.pone.0259635*

2. Furthermore, the authors chose to recategorize the wealth index variable. In Demographic and Health Surveys (DHS), this variable is originally constructed as quintiles, with households distributed approximately equally across five groups. However, the authors’ recategorization, combining the first two quintiles on one hand and the fourth and fifth quintiles on the other, resulted in an uneven distribution of observations across the resulting categories.

In this context, the rationale for referring to these categories as terciles is not respected. A more methodologically rigorous approach would have been to use the wealth index in its continuous form to generate true terciles based on the corresponding percentiles, rather than regrouping existing quintile categories.

**Authors’ response**

Thank you for your observation. We would like to clarify that our goal was not to construct statistical terciles based on the continuous wealth index score. Rather, the original DHS wealth quintiles were reclassified into three broader wealth categories: ‘Poor’ (combining the Poorest and Poorer quintiles), ‘Middle’, and ‘Rich’ (combining the Richer and Richest quintiles). This categorization was adopted from a previous WASH study by Dhital et al. (2022) and was used to facilitate interpretation of wealth-related disparities in WASH access and comparison of findings across survey rounds. As described in the Variables section (**page 6**), the five wealth index quintiles were reclassified into these three categories, and throughout the manuscript they are treated as broader wealth categories rather than statistical terciles. This categorization was applied consistently across all survey rounds to ensure comparability of findings over time.

***Reference*** *Dhital SR, Chojenta C, Evans TJ, Acharya TD, Loxton D. Prevalence and Correlates of Water, Sanitation, and Hygiene (WASH) and Spatial Distribution of Unimproved WASH in Nepal. Int J Environ Res Public Health. 2022;19(6). doi:10.3390/ijerph19063507.*

.
