# Supplementary material for: A decade of progress and challenges in Water, Sanitation and Hygiene (WASH) coverage in Bangladesh: Insights from Bangladesh demographic and health survey 2011–2022
Source: PLoS One. 2026 Jul 23;21(7):e0354480. doi: 10.1371/journal.pone.0354480 (PMC13395418; doi:10.1371/journal.pone.0354480)
Supplement: Peer review history

# Peer review: Decision Letter 0

**DOI:** 10.1371/journal.pone.0354480.r001

**Authors:** Rajib Chowdhury

17 Mar 2026

PONE-D-25-64861

A Decade of Progress and Challenges in Water, Sanitation and Hygiene (WASH) Coverage in Bangladesh: Insights from Bangladesh Demographic and Health Survey 2011-2022

PLOS One

Dear Dr. Jitu,

Thank you for submitting your manuscript to PLOS ONE. After careful consideration, we feel that it has merit but does not fully meet PLOS ONE’s publication criteria as it currently stands. Therefore, we invite you to submit a revised version of the manuscript that addresses the points raised during the review process.

Please submit your revised manuscript by May 01 2026 11:59PM. If you will need more time than this to complete your revisions, please reply to this message or contact the journal office at plosone@plos.org. When you're ready to submit your revision, log on to https://www.editorialmanager.com/pone/ and select the 'Submissions Needing Revision' folder to locate your manuscript file.

Please include the following items when submitting your revised manuscript:

- A letter that responds to each point raised by the academic editor and reviewer(s). You should upload this letter as a separate file labeled 'Response to Reviewers'.
- A marked-up copy of your manuscript that highlights changes made to the original version. You should upload this as a separate file labeled 'Revised Manuscript with Track Changes'.
- An unmarked version of your revised paper without tracked changes. You should upload this as a separate file labeled 'Manuscript'.

If you would like to make changes to your financial disclosure, please include your updated statement in your cover letter. Guidelines for resubmitting your figure files are available below the reviewer comments at the end of this letter.

If applicable, we recommend that you deposit your laboratory protocols in protocols.io to enhance the reproducibility of your results. Protocols.io assigns your protocol its own identifier (DOI) so that it can be cited independently in the future. For instructions see: https://journals.plos.org/plosone/s/submission-guidelines#loc-laboratory-protocols. Additionally, PLOS ONE offers an option for publishing peer-reviewed Lab Protocol articles, which describe protocols hosted on protocols.io. Read more information on sharing protocols at https://plos.org/protocols?utm_medium=editorial-email&utm_source=authorletters&utm_campaign=protocols.

We look forward to receiving your revised manuscript.

Kind regards,

Rajib Chowdhury, M.Sc.; MPH

Academic Editor

PLOS One

Journal Requirements:

When submitting your revision, we need you to address these additional requirements.

1. Please ensure that your manuscript meets PLOS ONE's style requirements, including those for file naming. The PLOS ONE style templates can be found at

https://journals.plos.org/plosone/s/file?id=wjVg/PLOSOne_formatting_sample_main_body.pdf and https://journals.plos.org/plosone/s/file?id=ba62/PLOSOne_formatting_sample_title_authors_affiliations.pdf

2. In the online submission form, you indicated that the secondary datasets analyzed in this study are available upon request from the Demographic and Health Surveys (DHS) Program. Access to the data requires registration and approval through the

DHS website: https://dhsprogram.com/data/ available-datasets. The authors do not have the authority to share the data directly.

All PLOS journals now require all data underlying the findings described in their manuscript to be freely available to other researchers, either 1. In a public repository, 2. Within the manuscript itself, or 3. Uploaded as supplementary information.

This policy applies to all data except where public deposition would breach compliance with the protocol approved by your research ethics board. If your data cannot be made publicly available for ethical or legal reasons (e.g., public availability would compromise patient privacy), please explain your reasons on resubmission and your exemption request will be escalated for approval.

3. Your ethics statement should only appear in the Methods section of your manuscript. If your ethics statement is written in any section besides the Methods, please delete it from any other section.

4. We note that Figure 4 in your submission contain [map/satellite] images which may be copyrighted. All PLOS content is published under the Creative Commons Attribution License (CC BY 4.0), which means that the manuscript, images, and Supporting Information files will be freely available online, and any third party is permitted to access, download, copy, distribute, and use these materials in any way, even commercially, with proper attribution. For these reasons, we cannot publish previously copyrighted maps or satellite images created using proprietary data, such as Google software (Google Maps, Street View, and Earth). For more information, see our copyright guidelines: http://journals.plos.org/plosone/s/licenses-and-copyright.

We require you to either (1) present written permission from the copyright holder to publish these figures specifically under the CC BY 4.0 license, or (2) remove the figures from your submission:

a. You may seek permission from the original copyright holder of Figure 4 to publish the content specifically under the CC BY 4.0 license.

We recommend that you contact the original copyright holder with the Content Permission Form (http://journals.plos.org/plosone/s/file?id=7c09/content-permission-form.pdf) and the following text:

“I request permission for the open-access journal PLOS ONE to publish XXX under the Creative Commons Attribution License (CCAL) CC BY 4.0 (http://creativecommons.org/licenses/by/4.0/). Please be aware that this license allows unrestricted use and distribution, even commercially, by third parties. Please reply and provide explicit written permission to publish XXX under a CC BY license and complete the attached form.”

Please upload the completed Content Permission Form or other proof of granted permissions as an "Other" file with your submission.

In the figure caption of the copyrighted figure, please include the following text: “Reprinted from [ref] under a CC BY license, with permission from [name of publisher], original copyright [original copyright year].”

b. If you are unable to obtain permission from the original copyright holder to publish these figures under the CC BY 4.0 license or if the copyright holder’s requirements are incompatible with the CC BY 4.0 license, please either i) remove the figure or ii) supply a replacement figure that complies with the CC BY 4.0 license. Please check copyright information on all replacement figures and update the figure caption with source information. If applicable, please specify in the figure caption text when a figure is similar but not identical to the original image and is therefore for illustrative purposes only.

The following resources for replacing copyrighted map figures may be helpful:

USGS National Map Viewer (public domain): http://viewer.nationalmap.gov/viewer/

The Gateway to Astronaut Photography of Earth (public domain): http://eol.jsc.nasa.gov/sseop/clickmap/

Maps at the CIA (public domain): https://www.cia.gov/library/publications/the-world-factbook/index.html and https://www.cia.gov/library/publications/cia-maps-publications/index.html

NASA Earth Observatory (public domain): http://earthobservatory.nasa.gov/

Landsat: http://landsat.visibleearth.nasa.gov/

USGS EROS (Earth Resources Observatory and Science (EROS) Center) (public domain): http://eros.usgs.gov/#

Natural Earth (public domain): http://www.naturalearthdata.com/

5. Please include captions for your Supporting Information files at the end of your manuscript, and update any in-text citations to match accordingly. Please see our Supporting Information guidelines for more information: http://journals.plos.org/plosone/s/supporting-information.

6. If the reviewer comments include a recommendation to cite specific previously published works, please review and evaluate these publications to determine whether they are relevant and should be cited. There is no requirement to cite these works unless the editor has indicated otherwise.

[Note: HTML markup is below. Please do not edit.]

Reviewers' comments:

Reviewer's Responses to Questions

**Comments to the Author**

1. Is the manuscript technically sound, and do the data support the conclusions?

The manuscript must describe a technically sound piece of scientific research with data that supports the conclusions. Experiments must have been conducted rigorously, with appropriate controls, replication, and sample sizes. The conclusions must be drawn appropriately based on the data presented.

Reviewer #1: Yes

Reviewer #2: Partly

**********

2. Has the statistical analysis been performed appropriately and rigorously?

Reviewer #1: Yes

Reviewer #2: Yes

**********

3. Have the authors made all data underlying the findings in their manuscript fully available?

The PLOS Data policy requires authors to make all data underlying the findings described in their manuscript fully available without restriction, with rare exception (please refer to the Data Availability Statement in the manuscript PDF file). The data should be provided as part of the manuscript or its supporting information, or deposited to a public repository. For example, in addition to summary statistics, the data points behind means, medians and variance measures should be available. If there are restrictions on publicly sharing data—e.g. participant privacy or use of data from a third party—those must be specified.

Reviewer #1: Yes

Reviewer #2: Yes

**********

4. Is the manuscript presented in an intelligible fashion and written in standard English?

PLOS ONE does not copyedit accepted manuscripts, so the language in submitted articles must be clear, correct, and unambiguous. Any typographical or grammatical errors should be corrected at revision, so please note any specific errors here.

Reviewer #1: Yes

Reviewer #2: No

**********

5. Review Comments to the Author

Please use the space provided to explain your answers to the questions above. You may also include additional comments for the author, including concerns about dual publication, research ethics, or publication ethics. (Please upload your review as an attachment if it exceeds 20,000 characters)

Reviewer #1: Abstract

1) Line 16-17: The first sentence is generic. I would expect at least one quantified global or Bangladesh‑specific burden estimate to anchor importance. Also, write the full abbreviation of WASH the 1st time you use it.

2) Line 22-25: It is not clear how “improved” WASH, sanitation, and hygiene are defined, nor whether the WHO/UNICEF JMP definitions are strictly followed and consistent across all four BDHS rounds. This should be explicit.

3) Authors mentioned spatial mapping, survey‑weighted logistic regression, GVIF, and classification trees. For an abstract, this is heavy on technical jargon; consider prioritising what is essential for understanding the findings.

4) Reporting “sanitation (22%), hygiene (30.5%), and overall WASH facilities (28%)” needs clarification: are these absolute percentage‑point increases or relative percentage changes? Please properly state that it is the percent change from 2011 to 2022.

5) Line 41-42: The statement “primary factors influencing WASH access have remained largely unchanged” is interesting but not supported with numerical evidence here. It would help to name those factors explicitly.

6) The policy recommendations are sound but generic. Please provide more specific policy recommendations.

Introduction

7) The introduction should more clearly situate Bangladesh in the global WASH and SDG‑6 landscape: How does its progress compare with regional neighbours or LMIC averages? At present, the text largely rehearses the importance of WASH but not Bangladesh’s relative performance. Please properly mention Bangladesh's standing in achieving the SDG 6 goals.

Methods

8) Line 171-73: Please mention whether descriptive statistics are weighted frequency and percentages.

9) Line 173-77: The authors correctly mentioned the use of survey‑weighted models. The methods should specify whether clustering and stratification were accounted for. Also, clarify whether analyses were done separately by survey round or as pooled data with year fixed effects.

10) Line 177-78: How was multicollinearity assessed using GVIF, and what decision rule (threshold) was applied?

11) Line 179-184: Please specify which outcome (overall WASH vs component) was modelled in the classification tree analysis. How you handled survey weights in tree building and how overfitting was addressed.

12) Line 185: For Spatial analysis, indicate whether you used smoothed estimates or raw weighted proportions. Clarify whether statistical tests for spatial clustering (like Moran’s I) were performed or if maps are descriptive only.

Results

13) I don’t understand why the authors provided the same information in Table 1 and Figure 3, and also in text lines 201-203. Please remove Table 1 and add the value labels in the figure.

14) As the main stated objective of this paper is to demonstrate decadal progress in WASH coverage, I strongly recommend that the authors add a table presenting the Average Annual Rate of Increase (AARI) for each WASH indicator. This should be shown both overall and stratified by key background characteristics. It will allow readers to see whether improvements have accelerated or stalled in specific subgroups.

15) Line 332-336: It does not add anything in result section. Please transfer the text in the methods and Table 4 as a supplementary table.

16) Line 341-350: The abstract mentioned that wealth is the most important split. This is unsurprising and already evident from regression. For the tree to justify inclusion, the Results should highlight novel high‑risk combinations (like “poorest quintile + large family size + no media access in specific divisions”), and quantify how much of the remaining unserved population is concentrated in these nodes.

17) As an optional but valuable addition, I encourage the authors to consider incorporating formal equity metrics, such as the concentration index and the slope index of inequality (SII), to quantify wealth‑ and education‑related gradients in WASH coverage over time.

Discussion

18) The Discussion should be restructured around a clear set of messages, like what magnitude of national progress has been achieved, by component, and to what extent have regional and wealth inequalities narrowed, persisted, or widened? What plausible policy or programmatic explanations exist? Please don’t repeat the result in this section.

19) The Discussion currently seems to restate the statistical associations (wealth, education, media access, phone ownership) without deeply exploring mechanisms or referring to implementation experience in Bangladesh (for example, roles of DPHE, NGO programs, national sanitation campaigns). Readers will expect linkage between numbers and real‑world systems.

20) Please provide a dedicated Policy Implication section before the conclusion. The recommended actions (“prioritize economically marginalised groups, subsidize WASH equipment, awareness campaigns”) are appropriate but broad. Consider being more concrete, like which subgroups, what types of investments, and how might the classification tree-identified high‑risk nodes be used operationally?

Reviewer #2: Major Comments

1. Novelty and Contribution

The manuscript claims (Lines 107–108, 428–429) that no study has comprehensively analyzed decade-long WASH progress using nationally representative data, or that this is the first such study. These statements are questionable. Several previous studies have used DHS, MICS, or similar datasets to examine WASH inequalities in Bangladesh.

Among others, the Bangladesh Health Watch publication “50 Years of Independence: Progress of Health Sector in Bangladesh” (University Press Limited, 2023) includes a dedicated chapter on WASH, where even 50 years of progress and challenges have been articulated.

The authors are encouraged to undertake a more comprehensive literature review, revisit these claims, and clearly articulate what additional knowledge this study contributes beyond the existing body of work.

2. Definitional Ambiguity and Incomplete Analysis

The manuscript relies on the term “improved WASH,” which was primarily used during the MDG era. In the SDG era (2015–2030), the monitoring framework has moved away from this terminology and uses service-level ladders. Continued use of “improved” without alignment to SDG definitions limits the usefulness of the findings for policy and programme purposes.

a) Drinking Water

Under the SDG framework, drinking water services are categorized as surface water, no service, unimproved, limited, basic, and safely managed. These indicators are central to SDG-6 monitoring, and their absence should be acknowledged as a limitation.

BDHS variables (e.g., Q103, Q107, Q108) allow at least partial classification into “basic” and “safely managed” services. The manuscript reports that access to improved drinking water exceeds 98% in Bangladesh (Line 203). However, under the WHO/UNICEF JMP framework, an “improved source” does not necessarily imply safe or safely managed drinking water.

In the Bangladesh context—where arsenic contamination and water quality remain significant concerns—this figure may overstate actual access to safe water. Furthermore, if collection time exceeds 30 minutes (Q103), the service cannot be classified even as “basic.” Therefore, such statements, if not properly qualified, may be misleading for policy interpretation. The authors should clarify this distinction or explicitly acknowledge it as a limitation.

b) Sanitation

Under the SDG framework, sanitation is classified as open defecation, unimproved, limited, basic, and safely managed. BDHS variables (Q110, Q111) capture shared sanitation, which is essential to distinguish between “limited” and “basic” services.

With additional analysis of the same dataset, it is possible to derive SDG-relevant sanitation service levels. The current analysis stops short of this, which limits its policy relevance.

c) Hygiene

In the SDG framework, hygiene is classified as no facility, limited, and basic. The term “improved hygiene” is not used. Therefore, the use of this terminology in the manuscript is not consistent with current global monitoring standards and should be revised.

Minor Comments

1. The descriptive results (Lines 250–251) suggest no notable differences in WASH coverage between male- and female-headed households. However, the regression results (Lines 304–305) indicate significantly higher odds of improved WASH access among female-headed households. The authors should clarify this apparent discrepancy and explain how adjustment for socioeconomic and demographic factors influences the observed relationship.

2. The manuscript applies classification tree analysis in addition to logistic regression models. However, the tree analysis appears to largely replicate the regression findings, particularly the dominant role of wealth in predicting WASH access. The authors should clarify the added analytical value of this approach or consider simplifying the analysis.

Interpretation of Findings

The discussion section largely reiterates statistical findings rather than providing deeper interpretation. For example, the strong association between wealth and WASH access is well established in the literature. The authors are encouraged to go beyond description and discuss why such inequalities persist despite improvements in coverage. Relevant factors may include amongst others are urban–rural infrastructure gaps, affordability constraints, governance, communication, access to TV and mobile phone, service delivery issues, and climate-related vulnerabilities.

Policy Implications

The policy recommendations are currently quite general. The manuscript would benefit from stronger linkage to specific national policies, sector strategies, or financing mechanisms in Bangladesh related to WASH and SDG implementation. This would enhance the practical relevance of the study.

Recommendation to the Editor

Decision: Major Revision

The manuscript addresses an important topic and uses a robust nationally representative dataset. However, substantial revisions are required. In particular, the authors should, to the extent possible, align the analysis with SDG-consistent WASH definitions; clarify where such alignment is not feasible; strengthen the articulation of novelty; improve methodological transparency; deepen interpretation of findings; and enhance policy relevance. Language and overall presentation also require improvement before the manuscript can be considered for publication.

**********

6. PLOS authors have the option to publish the peer review history of their article (what does this mean?). If published, this will include your full peer review and any attached files.

If you choose “no”, your identity will remain anonymous but your review may still be made public.

**Do you want your identity to be public for this peer review?**  For information about this choice, including consent withdrawal, please see our Privacy Policy.

Reviewer #1: **Yes:**  Syed Toukir Ahmed Noor

Reviewer #2: **Yes:**  Md Khairul Islam

**********

[NOTE: If reviewer comments were submitted as an attachment file, they will be attached to this email and accessible via the submission site. Please log into your account, locate the manuscript record, and check for the action link "View Attachments". If this link does not appear, there are no attachment files.]

To ensure your figures meet our technical requirements, please review our figure guidelines: https://journals.plos.org/plosone/s/figures

You may also use PLOS’s free figure tool, NAAS, to help you prepare publication quality figures: https://journals.plos.org/plosone/s/figures#loc-tools-for-figure-preparation.

NAAS will assess whether your figures meet our technical requirements by comparing each figure against our figure specifications.

Attachment Submitted filename: Comment to authors_PlosOne.pdf

# Author comment: Author response to Decision Letter 1

**DOI:** 10.1371/journal.pone.0354480.r002

12 May 2026

5 March 2026

Rajib Chowdhury

Academic Editor

PLOS ONE

RE: PLOS ONE Decision: Revision required [PONE-D-25-64861] - [EMID:e9ab643b302a71bf]

Dear Editor,

Thank you for proving us the opportunity to revise the paper titled “A Decade of Progress and Challenges in Water, Sanitation and Hygiene (WASH) Coverage in Bangladesh: Insights from Bangladesh Demographic and Health Survey 2011-2022” for publication in PLOS ONE.

We have addressed the reviewers’ comments in our responses and incorporated related changes into the original manuscript through a revision. For convenience, the reviewers’ comments are highlighted in blue, and our responses are in black font.

The primary changes made in the revised version of the manuscript include:

1. Revised methodology section clarified WASH indicator definitions, analytical framework, and comparability across survey rounds.

2. Revised results section improved clarity of findings, distinguished percentage point changes, incorporated the Average Annual Rate of Increase (AARI), and aligned descriptive and regression interpretations.

3. Revised discussion section restructured to reduce repetition and strengthened interpretation by incorporating underlying mechanisms and policy-relevant insights. A dedicated Policy Implications subsection has also been added.

In addition to the above, all authors have read the revised manuscript. Some minor changes throughout the revised version of the manuscript are made, mostly to improve clarity of expressions and correct grammatical errors.

We would like to thank you and reviewers for all the valuable feedback and suggestions.

Sincere regards,

The authors.

Title: A Decade of Progress and Challenges in Water, Sanitation and Hygiene (WASH) Coverage in Bangladesh: Insights from Bangladesh Demographic and Health Survey 2011-2022

Manuscript ID: PONE-D-25-64861

Journal Requirements:

1. Please ensure that your manuscript meets PLOS ONE's style requirements, including those for file naming. The PLOS ONE style templates can be found at

https://journals.plos.org/plosone/s/file?id=wjVg/PLOSOne_formatting_sample_main_body.pdf and https://journals.plos.org/plosone/s/file?id=ba62/PLOSOne_formatting_sample_title_authors_affiliations.pdf

Authors’ response

Thank you for the suggestion. We have ensured that our manuscript meets PLOS ONE's style requirements.

2. Please In the online submission form, you indicated that the secondary datasets analyzed in this study are available upon request from the Demographic and Health Surveys (DHS) Program. Access to the data requires registration and approval through the

DHS website: https://dhsprogram.com/data/ available-datasets. The authors do not have the authority to share the data directly.

All PLOS journals now require all data underlying the findings described in their manuscript to be freely available to other researchers, either 1. In a public repository, 2. Within the manuscript itself, or 3. Uploaded as supplementary information.

This policy applies to all data except where public deposition would breach compliance with the protocol approved by your research ethics board. If your data cannot be made publicly available for ethical or legal reasons (e.g., public availability would compromise patient privacy), please explain your reasons on resubmission and your exemption request will be escalated for approval.

Authors’ response

Thank you. We have revised the Data Availability statement to comply with the journal’s requirements and updated it in the “Data Availability” field of the submission form via the “Edit Submission” option.

“The data used in this study were obtained from the Demographic and Health Surveys (DHS) Program and constitute third-party data that are not owned by the authors. The Bangladesh Demographic and Health Survey (BDHS) datasets are available upon registration and approval through the DHS Program website (https://dhsprogram.com/data/available-datasets.cfm). The authors obtained access to the data through the standard request and approval process of the DHS Program and did not receive any special privileges or preferential access that would not be available to other researchers.”

3. Your ethics statement should only appear in the Methods section of your manuscript. If your ethics statement is written in any section besides the Methods, please delete it from any other section.

Authors’ response

Thank you for your comment. The ethics statement is already included under the “Ethical Considerations” subsection within the Methods section. We have ensured that it does not appear in any other section of the manuscript.

“Ethical Considerations

No ethical approval was required for this study as we utilized publicly available secondary data from BDHS, which had been already approved by institutional review boards (IRBs) at ICF and the Bangladesh Medical Research Council (BMRC). Additionally, written informed consent was obtained from participants before data collection, ensuring voluntary participation, confidentiality, and the right to withdraw at any time. The datasets were fully anonymized before access and are available upon request for research purposes through the DHS website (https://dhsprogram.com/).

” 4. We note that Figure 4 in your submission contain [map/satellite] images which may be copyrighted. All PLOS content is published under the Creative Commons Attribution License (CC BY 4.0), which means that the manuscript, images, and Supporting Information files will be freely available online, and any third party is permitted to access, download, copy, distribute, and use these materials in any way, even commercially, with proper attribution. For these reasons, we cannot publish previously copyrighted maps or satellite images created using proprietary data, such as Google software (Google Maps, Street View, and Earth). For more information, see our copyright guidelines: http://journals.plos.org/plosone/s/licenses-and-copyright.

We require you to either (1) present written permission from the copyright holder to publish these figures specifically under the CC BY 4.0 license, or (2) remove the figures from your submission:

a. You may seek permission from the original copyright holder of Figure 4 to publish the content specifically under the CC BY 4.0 license.

We recommend that you contact the original copyright holder with the Content Permission Form (http://journals.plos.org/plosone/s/file?id=7c09/content-permission-form.pdf) and the following text:

“I request permission for the open-access journal PLOS ONE to publish XXX under the Creative Commons Attribution License (CCAL) CC BY 4.0 (http://creativecommons.org/licenses/by/4.0/). Please be aware that this license allows unrestricted use and distribution, even commercially, by third parties. Please reply and provide explicit written permission to publish XXX under a CC BY license and complete the attached form.”

Please upload the completed Content Permission Form or other proof of granted permissions as an "Other" file with your submission.

In the figure caption of the copyrighted figure, please include the following text: “Reprinted from [ref] under a CC BY license, with permission from [name of publisher], original copyright [original copyright year].”

b. If you are unable to obtain permission from the original copyright holder to publish these figures under the CC BY 4.0 license or if the copyright holder’s requirements are incompatible with the CC BY 4.0 license, please either i) remove the figure or ii) supply a replacement figure that complies with the CC BY 4.0 license. Please check copyright information on all replacement figures and update the figure caption with source information. If applicable, please specify in the figure caption text when a figure is similar but not identical to the original image and is therefore for illustrative purposes only.

The following resources for replacing copyrighted map figures may be helpful:

USGS National Map Viewer (public domain): http://viewer.nationalmap.gov/viewer/

The Gateway to Astronaut Photography of Earth (public domain): http://eol.jsc.nasa.gov/sseop/clickmap/

Maps at the CIA (public domain): https://www.cia.gov/library/publications/the-world-factbook/index.html and https://www.cia.gov/library/publications/cia-maps-publications/index.html

NASA Earth Observatory (public domain): http://earthobservatory.nasa.gov/

Landsat: http://landsat.visibleearth.nasa.gov/

USGS EROS (Earth Resources Observatory and Science (EROS) Center) (public domain): http://eros.usgs.gov/#

Natural Earth (public domain): http://www.naturalearthdata.com/

Authors’ response

Apologies for any confusion. Figure 4 of our manuscript contain spatial mappings. This map is not copyrighted. This is original image created by us using the statistical software R. The R packages and shapefile used in this manuscript are listed on page 8; in the last paragraph of the Statistical analyses section. Besides, we did not use any R packages that source their maps from Google Maps. Instead, we utilized the administrative boundary shapefile of South Asian countries from an openly licensed database, the geoBoundaries Global Administrative Database (Runfola et al., 2020), which we have cited in the last paragraph of the Statistical analyses section.

Reference:

Runfola D, Anderson A, Baier H, Crittenden M, Dowker E, Fuhrig S, Goodman S, Grimsley G, Layko R, Melville G, Mulder M. geoBoundaries: A global database of political administrative boundaries. PloS one. 2020 Apr 24;15(4):e0231866.

5. Please include captions for your Supporting Information files at the end of your manuscript, and update any in-text citations to match accordingly. Please see our Supporting Information guidelines for more information: http://journals.plos.org/plosone/s/supporting-information.

Authors’ response

Thank you for raising the point. We have updated the manuscript accordingly and the following section has been added at the end of our manuscript (page 27 of the revised manuscript),

“Supporting information

S1 Table: Average Annual Rate of Increase (AARI) of improved WASH components in Bangladesh, 2011–2022.

S2 Table. GVIF for binary logistic regression model adjusted for demographic, socio-economic, and geographic factors with WASH facilities as outcome for BDHS 2011 and 2014.

S3 Table. GVIF for binary logistic regression model adjusted for demographic, socio-economic, and geographic factors with WASH facilities as outcome for BDHS 2017-18 and 2022.”

6. If the reviewer comments include a recommendation to cite specific previously published works, please review and evaluate these publications to determine whether they are relevant and should be cited. There is no requirement to cite these works unless the editor has indicated otherwise.

Authors’ response

Thank you for your comment. We have carefully reviewed the reviewer-suggested references and evaluated their relevance to our study. Relevant references have been incorporated into the revised manuscript where appropriate.

Reviewer #1

Reviewer’s comment

Abstract

1. Line 16-17: The first sentence is generic. I would expect at least one quantified global or Bangladesh specific burden estimate to anchor importance. Also, write the full abbreviation of WASH the 1st time you use it.

Authors’ response

Thank you for your comment. We have revised the background section of the abstract including opening sentence to define WASH and include quantitative evidence.

“Background

Access to safe water, sanitation, and hygiene (WASH) remains a critical global public health challenge, with an estimated 2 billion people lacking safe drinking water and basic hygiene services. In Bangladesh, disparities in WASH access persist across regions and socioeconomic groups. This study assesses decade-long changes in WASH coverage, examines its distribution across socio-demographic groups, identifies key associated factors, and highlights underserved regions and population subgroups.” (Page 1 of the revised Manuscript)

2. Line 22-25: It is not clear how “improved” WASH, sanitation, and hygiene are defined, nor whether the WHO/UNICEF JMP definitions are strictly followed and consistent across all four BDHS rounds. This should be explicit.

Authors’ response

Thank you for your comment. We have clarified in the Methods section of the abstract that WASH indicators were defined according to WHO–UNICEF Joint Monitoring Programme (JMP) 2017 guidelines and applied consistently across all survey rounds.

“Methods

Data from four rounds of the Bangladesh Demographic and Health Survey (BDHS) (2011–2022) were analyzed. WASH indicators were classified as improved or unimproved based on WHO–UNICEF Joint Monitoring Programme (JMP) 2017 guidelines and applied consistently across all survey rounds. Spatial mapping was used to assess regional disparities, while regression models identified factors associated with WASH facilities. A classification tree approach was used to explore interactions among predictors.” (Page 1 of the revised Manuscript)

3. Authors mentioned spatial mapping, survey weighted logistic regression, GVIF, and classification trees. For an abstract, this is heavy on technical jargon; consider prioritising what is essential for understanding the findings.

Authors’ response

Thank you for your comment. We have simplified the Methods section of the abstract by reducing technical details and retaining only the key analytical approaches, while preserving clarity and essential methodological information.

“Methods

Data from four rounds of the Bangladesh Demographic and Health Survey (BDHS) (2011–2022) were analyzed. WASH indicators were classified as improved or unimproved based on WHO–UNICEF Joint Monitoring Programme (JMP) 2017 guidelines and applied consistently across all survey rounds. Spatial mapping was used to assess regional disparities, while regression models identified factors associated with WASH facilities. A classification tree approach was used to explore interactions among predictors.” (Page 1 of the updated Manuscript)

4. Reporting “sanitation (22%), hygiene (30.5%), and overall WASH facilities (28%)” needs clarification: are these absolute percentage point increases or relative percentage changes? Please properly state that it is the percent change from 2011 to 2022.

Authors’ response

Thank you for your comment. We have clarified in the Results section of the abstract that the reported values represent absolute percentage point increases from 2011 to 2022.

“Over the decade (2011–2022), access to improved drinking water remained high (above 98%), with improvements in sanitation (22 percentage points), hygiene (30.5 percentage points), and overall WASH facilities (28 percentage points).”

5. Line 41-42: The statement “primary factors influencing WASH access have remained largely unchanged” is interesting but not supported with numerical evidence here. It would help to name those factors explicitly.

Authors’ response

Thank you for your comment. We have revised the conclusion to explicitly identify the key factors influencing WASH access, including wealth status, education level, and regional disparities.

“Key determinants of WASH access, including wealth, education, and regional disparities, remained largely unchanged over time.” (Page 1 of the updated Manuscript)

6. The policy recommendations are sound but generic. Please provide more specific policy recommendations.

Authors’ response

Thank you for your comment. We have refined the policy recommendations in the conclusion section of the abstract to make them more specific and actionable, including targeted subsidies for socioeconomically disadvantaged households and region-specific interventions in underserved areas.

“Achieving SDG 6 requires targeted subsidies for socioeconomically disadvantaged households, region-specific interventions in underserved areas such as Barishal and Mymensingh, and strengthened awareness programs.” (Page 1 of the updated Manuscript)

Introduction

7. The introduction should more clearly situate Bangladesh in the global WASH and SDG 6 landscape: How does its progress compare with regional neighbours

Attachment Submitted filename: Response to Reviewers.docx

# Peer review: Decision Letter 1

**DOI:** 10.1371/journal.pone.0354480.r003

**Authors:** Rajib Chowdhury

22 Jun 2026

PONE-D-25-64861R1

A Decade of Progress and Challenges in Water, Sanitation and Hygiene (WASH) Coverage in Bangladesh: Insights from Bangladesh Demographic and Health Survey 2011-2022

PLOS One

Dear Dr. Jitu,

Thank you for submitting your manuscript to PLOS ONE. After careful consideration, we feel that it has merit but does not fully meet PLOS ONE’s publication criteria as it currently stands. Therefore, we invite you to submit a revised version of the manuscript that addresses the points raised during the review process.

Please submit your revised manuscript by Aug 06 2026 11:59PM. If you will need more time than this to complete your revisions, please reply to this message or contact the journal office at plosone@plos.org. When you're ready to submit your revision, log on to https://www.editorialmanager.com/pone/ and select the 'Submissions Needing Revision' folder to locate your manuscript file.

Please include the following items when submitting your revised manuscript:

- A letter that responds to each point raised by the academic editor and reviewer(s). You should upload this letter as a separate file labeled 'Response to Reviewers'.
- A marked-up copy of your manuscript that highlights changes made to the original version. You should upload this as a separate file labeled 'Revised Manuscript with Track Changes'.
- An unmarked version of your revised paper without tracked changes. You should upload this as a separate file labeled 'Manuscript'.

If you would like to make changes to your financial disclosure, please include your updated statement in your cover letter. Guidelines for resubmitting your figure files are available below the reviewer comments at the end of this letter.

If applicable, we recommend that you deposit your laboratory protocols in protocols.io to enhance the reproducibility of your results. Protocols.io assigns your protocol its own identifier (DOI) so that it can be cited independently in the future. For instructions see: https://journals.plos.org/plosone/s/submission-guidelines#loc-laboratory-protocols. Additionally, PLOS ONE offers an option for publishing peer-reviewed Lab Protocol articles, which describe protocols hosted on protocols.io. Read more information on sharing protocols at https://plos.org/protocols?utm_medium=editorial-email&utm_source=authorletters&utm_campaign=protocols.

As the corresponding author, your ORCID iD is verified in the submission system and will appear in the published article. PLOS supports the use of ORCID, and we encourage all coauthors to register for an ORCID iD and use it as well. Please encourage your coauthors to verify their ORCID iD within the submission system before final acceptance, as unverified ORCID iDs will not appear in the published article. *Only*  the individual author can complete the verification step; PLOS staff *cannot*  verify ORCID iDs on behalf of authors.

We look forward to receiving your revised manuscript.

Kind regards,

Rajib Chowdhury, M.Sc.; MPH

Academic Editor

PLOS One

Journal Requirements:

If the reviewer comments include a recommendation to cite specific previously published works, please review and evaluate these publications to determine whether they are relevant and should be cited. There is no requirement to cite these works unless the editor has indicated otherwise.

[Note: HTML markup is below. Please do not edit.]

Reviewers' comments:

Reviewer's Responses to Questions

**Comments to the Author**

1. If the authors have adequately addressed your comments raised in a previous round of review and you feel that this manuscript is now acceptable for publication, you may indicate that here to bypass the “Comments to the Author” section, enter your conflict of interest statement in the “Confidential to Editor” section, and submit your "Accept" recommendation.

Reviewer #1: All comments have been addressed

Reviewer #2: (No Response)

Reviewer #3: (No Response)

**********

2. Is the manuscript technically sound, and do the data support the conclusions?

The manuscript must describe a technically sound piece of scientific research with data that supports the conclusions. Experiments must have been conducted rigorously, with appropriate controls, replication, and sample sizes. The conclusions must be drawn appropriately based on the data presented.

Reviewer #1: Yes

Reviewer #2: (No Response)

Reviewer #3: Yes

**********

3. Has the statistical analysis been performed appropriately and rigorously?

Reviewer #1: Yes

Reviewer #2: (No Response)

Reviewer #3: Yes

**********

4. Have the authors made all data underlying the findings in their manuscript fully available?

The PLOS Data policy requires authors to make all data underlying the findings described in their manuscript fully available without restriction, with rare exception (please refer to the Data Availability Statement in the manuscript PDF file). The data should be provided as part of the manuscript or its supporting information, or deposited to a public repository. For example, in addition to summary statistics, the data points behind means, medians and variance measures should be available. If there are restrictions on publicly sharing data—e.g. participant privacy or use of data from a third party—those must be specified.

Reviewer #1: Yes

Reviewer #2: (No Response)

Reviewer #3: Yes

**********

5. Is the manuscript presented in an intelligible fashion and written in standard English?

PLOS ONE does not copyedit accepted manuscripts, so the language in submitted articles must be clear, correct, and unambiguous. Any typographical or grammatical errors should be corrected at revision, so please note any specific errors here.

Reviewer #1: Yes

Reviewer #2: (No Response)

Reviewer #3: Yes

**********

6. Review Comments to the Author

Please use the space provided to explain your answers to the questions above. You may also include additional comments for the author, including concerns about dual publication, research ethics, or publication ethics. (Please upload your review as an attachment if it exceeds 20,000 characters)

Reviewer #1: The revision addresses my comments well overall. Good luck. For the classification tree, I would still encourage the authors to report, for the lowest‑coverage terminal nodes, the proportion of all households lacking improved WASH that fall into these nodes, to better quantify how concentrated unmet need is within the identified high‑risk subgroups.

Reviewer #2: (No Response)

Reviewer #3: I am honored to participate in this review. Below are several comments intended to strengthen the manuscript.

Comment 1

This first concern had already been raised by previous reviewers but does not appear to have been adequately addressed. The terminology “improved” versus “unimproved” corresponds to the classification used under the Millennium Development Goals (MDGs), which remained in effect until 2015. Since the adoption of the Sustainable Development Goals (SDGs), water, sanitation, and hygiene (WASH) services have been classified into the following categories: safely managed, basic, limited, unimproved, and no service.

Based on the definitions adopted by the authors, the water sources categorized as “improved” in this study more closely correspond to “limited” water services under the SDG framework. Indeed, SDG limited water services include sources such as piped water into a dwelling, yard, or plot, public standpipes, boreholes or tube wells, protected dug wells, protected springs, rainwater, tanker trucks, carts with tanks, and bottled water. However, this classification does not take into account the time required to collect water. In contrast, a basic water service requires not only the use of an improved source but also that the total round-trip time for collecting water does not exceed 30 minutes.

Basic sanitation services refer to the use of improved sanitation facilities, including flush or pour-flush toilets connected to a sewer system, septic tank, or pit latrine, improved pit latrines with slabs, and composting toilets. However, this category includes an additional requirement: the facility must not be shared with other households. By contrast, limited sanitation services refer to the same types of improved facilities when they are shared by two or more households. Therefore, the distinction between “basic” and “limited” sanitation services is based not on the type of facility used, but rather on whether its use is exclusive or shared.

Basic hygiene facilities are handwashing facilities with soap and water available on the household premises.

Consequently, the study’s focus on limited water and sanitation services appears misaligned with the SDG programmatic targets. SDG 1 aims to end poverty in all its forms and includes Target 1.4, which calls for universal access to basic services, particularly for poor and vulnerable populations.

Comment 2

Furthermore, the authors chose to recategorize the wealth index variable. In Demographic and Health Surveys (DHS), this variable is originally constructed as quintiles, with households distributed approximately equally across five groups. However, the authors’ recategorization, combining the first two quintiles on one hand and the fourth and fifth quintiles on the other, resulted in an uneven distribution of observations across the resulting categories.

In this context, the rationale for referring to these categories as terciles is not respected. A more methodologically rigorous approach would have been to use the wealth index in its continuous form to generate true terciles based on the corresponding percentiles, rather than regrouping existing quintile categories.

**********

7. PLOS authors have the option to publish the peer review history of their article (what does this mean?). If published, this will include your full peer review and any attached files.

If you choose “no”, your identity will remain anonymous but your review may still be made public.

**Do you want your identity to be public for this peer review?**  For information about this choice, including consent withdrawal, please see our Privacy Policy.

Reviewer #1: **Yes:**  Syed Toukir Ahmed Noor

Reviewer #2: **Yes:**  Md Khairul Islam

Reviewer #3: No

**********

[NOTE: If reviewer comments were submitted as an attachment file, they will be attached to this email and accessible via the submission site. Please log into your account, locate the manuscript record, and check for the action link "View Attachments". If this link does not appear, there are no attachment files.]

To ensure your figures meet our technical requirements, please review our figure guidelines: https://journals.plos.org/plosone/s/figures

You may also use PLOS’s free figure tool, NAAS, to help you prepare publication quality figures: https://journals.plos.org/plosone/s/figures#loc-tools-for-figure-preparation.

NAAS will assess whether your figures meet our technical requirements by comparing each figure against our figure specifications.

# Author comment: Author response to Decision Letter 2

**DOI:** 10.1371/journal.pone.0354480.r004

22 Jun 2026

22 June 2026

Rajib Chowdhury

Academic Editor

PLOS ONE

RE: PLOS ONE Decision: Revision required [PONE-D-25-64861R1] - [EMID:5b1a1b4a25a68fe2]

Dear Editor,

Thank you for providing us the opportunity to revise the paper titled “A Decade of Progress and Challenges in Water, Sanitation and Hygiene (WASH) Coverage in Bangladesh: Insights from Bangladesh Demographic and Health Survey 2011–2022” for publication in PLOS ONE.

We have addressed the reviewers’ comments in our responses. For convenience, the reviewers’ comments are highlighted in blue, and our responses are presented in black font.

No substantive changes were made to the manuscript in this revision. The reviewers’ comments were addressed through clarification in the response document. The only changes to the manuscript relate to minor updates in reference numbering resulting from the revision process.

We would like to thank the editor and reviewers for their valuable feedback and constructive suggestions.

Sincere regards,

The Authors

Title: A Decade of Progress and Challenges in Water, Sanitation and Hygiene (WASH) Coverage in Bangladesh: Insights from Bangladesh Demographic and Health Survey 2011-2022

Manuscript ID: PONE-D-25-64861R1

Journal Requirements:

1. If the reviewer comments include a recommendation to cite specific previously published works, please review and evaluate these publications to determine whether they are relevant and should be cited. There is no requirement to cite these works unless the editor has indicated otherwise.

Authors’ response

Thank you for your comment. We have carefully reviewed the reviewer-suggested references and evaluated their relevance to our study. Relevant references have been incorporated into the revised manuscript where appropriate.

Reviewer #1

Reviewer’s comment

1. The revision addresses my comments well overall. Good luck. For the classification tree, I would still encourage the authors to report, for the lowest coverage terminal nodes, the proportion of all households lacking improved WASH that fall into these nodes, to better quantify how concentrated unmet need is within the identified high risk subgroups.

Authors’ response

Thank you for your suggestion. We agree that quantifying the proportion of households lacking improved WASH that are concentrated within the lowest-coverage terminal nodes would provide additional insight into the distribution of unmet need. However, the primary objective of the classification tree analysis in this study was to identify high-risk population subgroups and the interactions among key predictors associated with WASH access, rather than to quantify the concentration of unmet need across population segments. We therefore retained the current presentation focused on vulnerable population profiles. We appreciate this suggestion and recognize it as a valuable direction for future research.

Reviewer #3

Reviewer’s comment

1. This first concern had already been raised by previous reviewers but does not appear to have been adequately addressed. The terminology “improved” versus “unimproved” corresponds to the classification used under the Millennium Development Goals (MDGs), which remained in effect until 2015. Since the adoption of the Sustainable Development Goals (SDGs), water, sanitation, and hygiene (WASH) services have been classified into the following categories: safely managed, basic, limited, unimproved, and no service.

Based on the definitions adopted by the authors, the water sources categorized as “improved” in this study more closely correspond to “limited” water services under the SDG framework. Indeed, SDG limited water services include sources such as piped water into a dwelling, yard, or plot, public standpipes, boreholes or tube wells, protected dug wells, protected springs, rainwater, tanker trucks, carts with tanks, and bottled water. However, this classification does not take into account the time required to collect water. In contrast, a basic water service requires not only the use of an improved source but also that the total round-trip time for collecting water does not exceed 30 minutes.

Basic sanitation services refer to the use of improved sanitation facilities, including flush or pour-flush toilets connected to a sewer system, septic tank, or pit latrine, improved pit latrines with slabs, and composting toilets. However, this category includes an additional requirement: the facility must not be shared with other households. By contrast, limited sanitation services refer to the same types of improved facilities when they are shared by two or more households. Therefore, the distinction between “basic” and “limited” sanitation services is based not on the type of facility used, but rather on whether its use is exclusive or shared.

Basic hygiene facilities are handwashing facilities with soap and water available on the household premises.

Consequently, the study’s focus on limited water and sanitation services appears misaligned with the SDG programmatic targets. SDG 1 aims to end poverty in all its forms and includes Target 1.4, which calls for universal access to basic services, particularly for poor and vulnerable populations.

Authors’ response

Thank you for your comment. We agree that the SDG framework classifies water, sanitation, and hygiene services using service-level ladders (safely managed, basic, limited, unimproved, and no service), which differ from the traditional WHO–UNICEF JMP improved/unimproved classifications. We also acknowledge that the distinction between basic and limited services depends on additional criteria, such as collection time for drinking water and sharing status for sanitation facilities.

We would like to note that a similar concern was raised by a previous reviewer and, in response, we revised the manuscript to clarify the rationale for adopting the WHO–UNICEF JMP improved/unimproved classification framework. The primary objective of this study was not to generate official SDG service-level indicators or SDG monitoring estimates. Rather, as stated in the Introduction (Page 5):

“To address this gap, this study aims to assess decade-long changes in WASH coverage, examine its distribution across socio-demographic groups, identify key factors associated with WASH access, and highlight underserved regions and population subgroups using four rounds of nationally representative Bangladesh Demographic and Health Surveys (BDHS) data from 2011 to 2022.”

To achieve this objective, it was essential to apply a consistent classification framework across all four BDHS survey rounds. Therefore, WASH indicators were classified according to the WHO–UNICEF JMP improved/unimproved definitions and applied uniformly throughout the study period. This approach enabled meaningful assessment of temporal changes, socio-demographic inequalities, associated factors, and high-risk population subgroups over the 2011–2022 period.

We have also explicitly acknowledged this limitation in the revised manuscript (Discussion section, Page 25), where we state:

“The classification of WASH indicators in this study is based on the WHO–UNICEF Joint Monitoring Programme (JMP) ‘improved’ definitions to ensure comparability across survey rounds; however, these do not directly correspond to SDG service-level classifications (e.g., basic or safely managed), and may therefore overestimate actual access to safe services, particularly in Bangladesh, where issues such as water quality (e.g., arsenic contamination) and accessibility constraints remain important.”

Therefore, while SDG service-level classifications are important for global monitoring and programmatic evaluation, the use of the JMP improved/unimproved framework was necessary to achieve the objectives of the present study and ensure methodological consistency and comparability across all BDHS survey rounds. Furthermore, similar nationally representative studies conducted in Bangladesh have adopted the WHO–UNICEF JMP improved/unimproved classification framework to assess WASH coverage, inequalities, and associated factors, supporting its continued utility for population-level trend and disparity analyses (Ahmed et al., 2021).

Reference

Ahmed MS, Islam MI, Das MC, Khan A, Yunus FM. Mapping and situation analysis of basic WASH facilities at households in Bangladesh: Evidence from a nationally representative survey. PLoS ONE. 2021. doi:10.1371/journal.pone.0259635

2. Furthermore, the authors chose to recategorize the wealth index variable. In Demographic and Health Surveys (DHS), this variable is originally constructed as quintiles, with households distributed approximately equally across five groups. However, the authors’ recategorization, combining the first two quintiles on one hand and the fourth and fifth quintiles on the other, resulted in an uneven distribution of observations across the resulting categories.

In this context, the rationale for referring to these categories as terciles is not respected. A more methodologically rigorous approach would have been to use the wealth index in its continuous form to generate true terciles based on the corresponding percentiles, rather than regrouping existing quintile categories.

Authors’ response

Thank you for your observation. We would like to clarify that our goal was not to construct statistical terciles based on the continuous wealth index score. Rather, the original DHS wealth quintiles were reclassified into three broader wealth categories: ‘Poor’ (combining the Poorest and Poorer quintiles), ‘Middle’, and ‘Rich’ (combining the Richer and Richest quintiles). This categorization was adopted from a previous WASH study by Dhital et al. (2022) and was used to facilitate interpretation of wealth-related disparities in WASH access and comparison of findings across survey rounds. As described in the Variables section (page 6), the five wealth index quintiles were reclassified into these three categories, and throughout the manuscript they are treated as broader wealth categories rather than statistical terciles. This categorization was applied consistently across all survey rounds to ensure comparability of findings over time.

Reference

Dhital SR, Chojenta C, Evans TJ, Acharya TD, Loxton D. Prevalence and Correlates of Water, Sanitation, and Hygiene (WASH) and Spatial Distribution of Unimproved WASH in Nepal. Int J Environ Res Public Health. 2022;19(6). doi:10.3390/ijerph19063507.

Attachment Submitted filename: Response_to_Reviewers_auresp_2.docx

# Peer review: Decision Letter 2

**DOI:** 10.1371/journal.pone.0354480.r005

**Authors:** Rajib Chowdhury

8 Jul 2026

A Decade of Progress and Challenges in Water, Sanitation and Hygiene (WASH) Coverage in Bangladesh: Insights from Bangladesh Demographic and Health Survey 2011-2022

PONE-D-25-64861R2

Dear Dr. Jitu,

We’re pleased to inform you that your manuscript has been judged scientifically suitable for publication and will be formally accepted for publication once it meets all outstanding technical requirements.

Within one week, you’ll receive an e-mail detailing the required amendments. When these have been addressed, you’ll receive a formal acceptance letter and your manuscript will be scheduled for publication.

An invoice will be generated when your article is formally accepted. Please note, if your institution has a publishing partnership with PLOS and your article meets the relevant criteria, all or part of your publication costs will be covered. Please make sure your user information is up-to-date by logging into Editorial Manager at Editorial Manager® and clicking the ‘Update My Information' link at the top of the page. For questions related to billing, please contact billing support.

If your institution or institutions have a press office, please notify them about your upcoming paper to help maximize its impact. If they’ll be preparing press materials, please inform our press team as soon as possible -- no later than 48 hours after receiving the formal acceptance. Your manuscript will remain under strict press embargo until 2 pm Eastern Time on the date of publication. For more information, please contact onepress@plos.org.

Kind regards,

Rajib Chowdhury, M.Sc.; MPH

Academic Editor

PLOS One

Additional Editor Comments (optional):

Reviewers' comments:

Reviewer's Responses to Questions

**Comments to the Author**

1. If the authors have adequately addressed your comments raised in a previous round of review and you feel that this manuscript is now acceptable for publication, you may indicate that here to bypass the “Comments to the Author” section, enter your conflict of interest statement in the “Confidential to Editor” section, and submit your "Accept" recommendation.

Reviewer #1: All comments have been addressed

Reviewer #2: (No Response)

Reviewer #3: All comments have been addressed

**********

2. Is the manuscript technically sound, and do the data support the conclusions?

The manuscript must describe a technically sound piece of scientific research with data that supports the conclusions. Experiments must have been conducted rigorously, with appropriate controls, replication, and sample sizes. The conclusions must be drawn appropriately based on the data presented.

Reviewer #1: Yes

Reviewer #2: (No Response)

Reviewer #3: Yes

**********

3. Has the statistical analysis been performed appropriately and rigorously?

Reviewer #1: Yes

Reviewer #2: (No Response)

Reviewer #3: Yes

**********

4. Have the authors made all data underlying the findings in their manuscript fully available?

The PLOS Data policy requires authors to make all data underlying the findings described in their manuscript fully available without restriction, with rare exception (please refer to the Data Availability Statement in the manuscript PDF file). The data should be provided as part of the manuscript or its supporting information, or deposited to a public repository. For example, in addition to summary statistics, the data points behind means, medians and variance measures should be available. If there are restrictions on publicly sharing data—e.g. participant privacy or use of data from a third party—those must be specified.

Reviewer #1: Yes

Reviewer #2: (No Response)

Reviewer #3: Yes

**********

5. Is the manuscript presented in an intelligible fashion and written in standard English?

PLOS ONE does not copyedit accepted manuscripts, so the language in submitted articles must be clear, correct, and unambiguous. Any typographical or grammatical errors should be corrected at revision, so please note any specific errors here.

Reviewer #1: Yes

Reviewer #2: (No Response)

Reviewer #3: Yes

**********

6. Review Comments to the Author

Please use the space provided to explain your answers to the questions above. You may also include additional comments for the author, including concerns about dual publication, research ethics, or publication ethics. (Please upload your review as an attachment if it exceeds 20,000 characters)

Reviewer #1: (No Response)

Reviewer #2: (No Response)

Reviewer #3: Upon review of the submitted manuscript, several comments were raised. The authors have addressed them satisfactorily by providing the necessary clarifications.

**********

7. PLOS authors have the option to publish the peer review history of their article (what does this mean?). If published, this will include your full peer review and any attached files.

If you choose “no”, your identity will remain anonymous but your review may still be made public.

**Do you want your identity to be public for this peer review?**  For information about this choice, including consent withdrawal, please see our Privacy Policy.

Reviewer #1: No

Reviewer #2: **Yes:**  Md Khairul Islam

Reviewer #3: No

**********

# Editor report: Acceptance letter

**DOI:** 10.1371/journal.pone.0354480.r006

**Authors:** Rajib Chowdhury

PONE-D-25-64861R2

PLOS One

Dear Dr. Jitu,

I'm pleased to inform you that your manuscript has been deemed suitable for publication in PLOS One. Congratulations! Your manuscript is now being handed over to our production team.

At this stage, our production department will prepare your paper for publication. This includes ensuring the following:

* All references, tables, and figures are properly cited

* All relevant supporting information is included in the manuscript submission,

* There are no issues that prevent the paper from being properly typeset

You will receive further instructions from the production team, including instructions on how to review your proof when it is ready. Please keep in mind that we are working through a large volume of accepted articles, so please give us a few days to review your paper and let you know the next and final steps.

Lastly, if your institution or institutions have a press office, please let them know about your upcoming paper now to help maximize its impact. If they'll be preparing press materials, please inform our press team within the next 48 hours. Your manuscript will remain under strict press embargo until 2 pm Eastern Time on the date of publication. For more information, please contact onepress@plos.org.

You will receive an invoice from PLOS for your publication fee after your manuscript has reached the completed accept phase. If you receive an email requesting payment before acceptance or for any other service, this may be a phishing scheme. Learn how to identify phishing emails and protect your accounts at https://explore.plos.org/phishing.

If we can help with anything else, please email us at customercare@plos.org.

Thank you for submitting your work to PLOS ONE and supporting open access.

Kind regards,

PLOS ONE Editorial Office Staff

on behalf of

Dr. Rajib Chowdhury

Academic Editor

PLOS One
